# Beyond BMI: The Impact of the New Lancet Commission Diagnostic Criteria on Prevalence of Obesity in the United States

**DOI:** 10.1002/oby.70144

**Published:** 2026-02-22

**Authors:** Jennifer H. Hwang, Neda Laiteerapong

**Affiliations:** ^1^ Section of General Internal Medicine, Department of Medicine University of Chicago Chicago Illinois USA; ^2^ Department of Psychiatry and Behavioral Neuroscience University of Chicago Chicago Illinois USA

**Keywords:** body mass index, excess adiposity, Lancet Commission, obesity

## Abstract

**Objective:**

This study compared population characteristics and overall US prevalence of obesity over time using the BMI criteria and the 2025 Lancet Commission criteria (i.e., the Excess Adiposity criteria).

**Methods:**

We analyzed data from 58,053 adults aged ≥ 20 years in the National Health and Nutrition Examination Survey from 1999 to 2023. The Excess Adiposity criteria incorporated BMI, waist circumference, waist to hip and waist to height ratios, and body fat percentage to identify obesity, which was subclassified as preclinical or clinical based on organ dysfunction and activity limitations. Survey‐weighted estimates were calculated to produce nationally representative trends.

**Results:**

By BMI criteria, obesity prevalence increased from 31% (58.8 million) in 1999–2004 to 43% (101.3 million) in 2021–2023. By Excess Adiposity criteria, prevalence was higher and increasing from 51% (94.7 million) to 61% (143.9 million). The prevalence of clinical obesity is high due to a large number of people meeting criteria for organ dysfunction and/or activity limitations.

**Conclusions:**

Using the Excess Adiposity criteria to define obesity classifies nearly three in five US adults as having obesity, revealing substantial clinical, public health, and policy implications. The new criteria improve identification of individuals at risk but will require further strategies to achieve equitable, effective, and sustainable obesity prevention and treatment.

## Study Importance

- What is already known?
  ○ BMI does not capture body fat distribution, obesity‐associated organ dysfunction and functional impairment, or racial and ethnic variation in metabolic risk.
  ○ The 2025 Lancet Commission introduced a new framework, but its impact on US population trends is unknown.
- What does this study add?
  ○ By BMI criteria, obesity prevalence increased from 31% (58.8 million) in 1999–2004 to 43% (101.3 million) in 2021–2023. By Excess Adiposity criteria, prevalence was higher and increased from 50% (94.7 million) in 1999–2004 to 61% (143.9 million) in 2021–2023.
  ○ The prevalence of clinical obesity increased from 45% in 1999–2004 to 55% in 2021–2023 due to a large number of people meeting criteria for organ dysfunction and/or activity limitations.
- How might these results change the direction of research or the focus of clinical practice?
  ○ Findings reveal that BMI substantially underestimates obesity prevalence and associated dysfunction.
  ○ Including the excess adiposity criteria in definitions of obesity may improve early detection and treatment of obesity.

## 1 Introduction

Since the American Medical Association (AMA) recognized obesity as a disease in 2013, clinical and policy frameworks have evolved, emphasizing the need for effective prevention and treatment strategies [1]. Traditionally, body mass index (BMI) has been the cornerstone of obesity definitions worldwide, serving as a tool to identify individuals at risk of obesity‐associated complications [2, 3]. However, BMI‐based criteria (hereafter, BMI criteria) for defining obesity have several limitations: (1) they do not distinguish between fat and lean mass, (2) they fail to account for variations in body fat distribution, (3) they provide no direct insight into the functional impact of excess adiposity on organ systems or daily activities, and (4) they do not accurately reflect the risk of developing conditions such as hypertension, diabetes, and hyperlipidemia in certain racial and ethnic groups (e.g., non‐Hispanic Asians), who experience these complications at lower BMI levels [2, 4, 5, 6].

Given these limitations, alternative measures such as waist circumference (WC), waist to hip ratio (WHR), and waist to height ratio (WHtR) have been proposed to address BMI's shortcomings, but these metrics have their own limitations [7]. They often fail to accurately reflect subcutaneous and visceral fat compositions and are influenced by sex, age, and racial and ethnic differences [8]. Therefore, these tools alone remain inadequate for reliably diagnosing obesity and guiding treatment strategies. These limitations can lead to both underestimation and overestimation of adiposity‐associated disease burden, which impacts clinical practice, eligibility for insurance coverage of obesity treatments, and health policy decisions.

In 2025, the Lancet Commission, comprising 58 global experts, proposed a new definition and classification of obesity to address these limitations [9]. This framework shifts away from BMI criteria to a functionally driven approach that aligns with other chronic disease frameworks [9]. In addition to BMI, it identifies excess adiposity using body measurements (i.e., WC, WHR, WHtR) and direct body fat measurement, and it includes obesity‐associated dysfunction and activity limitations.

Building on recent studies [10, 11, 12, 13] that applied the Lancet Commission's framework to US adults, we examined national trends in obesity and population characteristics while comparing BMI‐defined and excess adiposity–defined obesity. By applying these new criteria, we estimate individuals who may have been previously excluded from treatment under BMI criteria despite having obesity‐associated functional impairment or elevated health risks. We also assessed sociodemographic, anthropometric, and clinical characteristics to determine whether individuals identified by the new criteria exhibit greater metabolic risk or disease burden than those classified by BMI alone. These findings have critical implications for treatment eligibility and accessibility, particularly for metabolic surgery and the new era of highly effective pharmacotherapy (e.g., glucagon‐like peptide‐1 receptor agonists [GLP‐1RA]).

## 2 Methods

### 2.1 Study Design and Source

**FIGURE 1:**
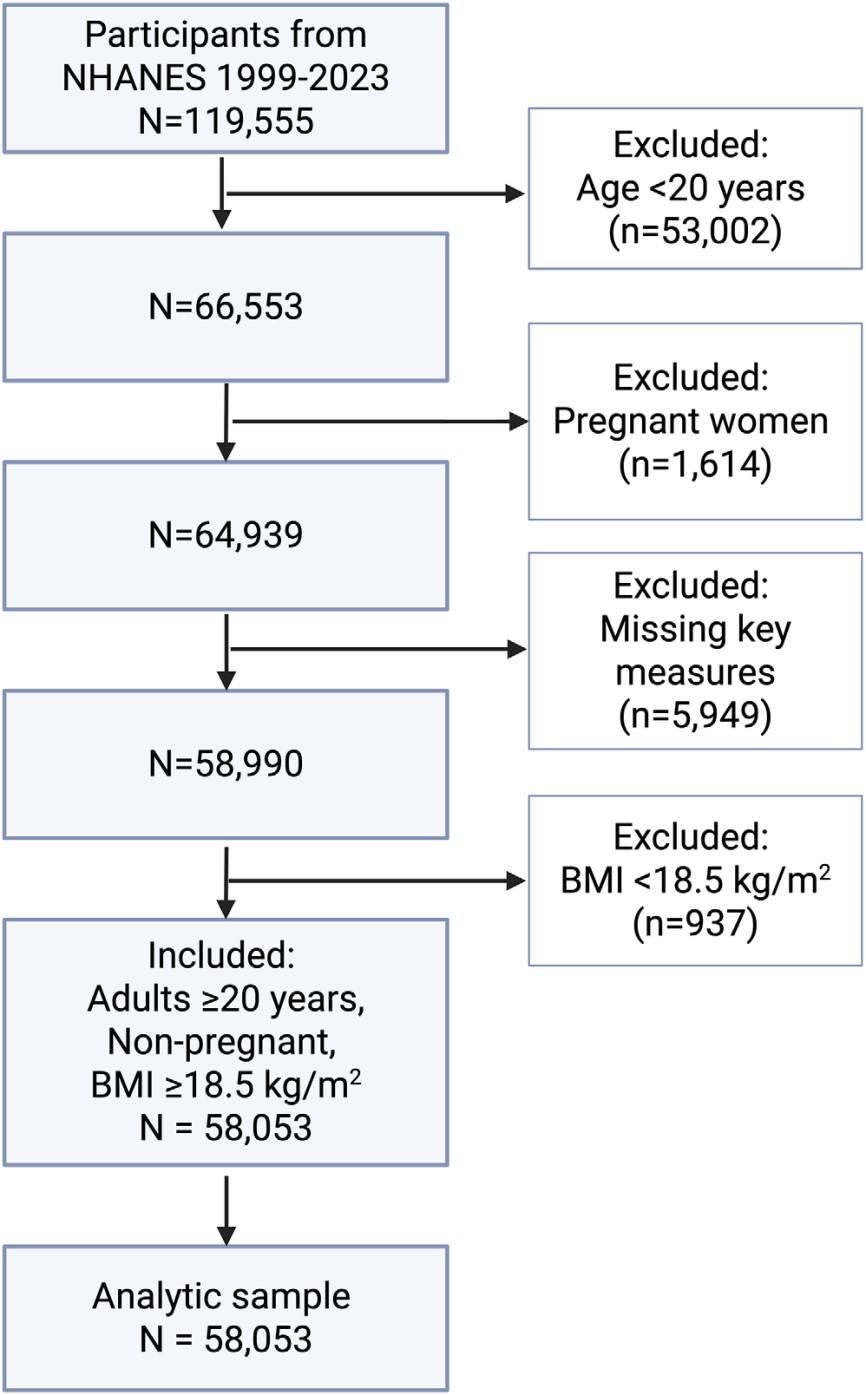
Flowchart of the study. NHANES, National Health and Nutrition Examination Survey. [Color figure can be viewed at wileyonlinelibrary.com]

This study analyzed National Health and Nutrition Examination Survey (NHANES) data from 1999 to 2023. NHANES is a nationally representative, cross‐sectional survey designed to assess the health of the US noninstitutionalized population. We included adults aged ≥ 20 years from each survey cycle and excluded pregnant participants and those with BMI < 18.5 kg/m^2^. The analytic sample comprised 58,053 adults, representing 12,752 adults (188 million US adults) in 1999–2004, 15,572 (205 million) in 2005–2010, 15,708 (221 million) in 2011–2016, 8174 (232 million) in 2017–2020, and 5847 (236 million) in 2021–2023 (Figure 1). The University of Chicago institutional review board determined this study was not human subjects research and did not require review. The study adhered to the Strengthening the Reporting of Observational Studies in Epidemiology (STROBE) reporting guideline [14].

### 2.2 Definitions of Obesity for BMI Criteria and the Excess Obesity Criteria

Obesity prevalence was estimated using two definitions: (1) BMI criteria and (2) the Lancet Commission criteria (hereafter, the Excess Adiposity criteria). BMI was calculated as weight in kilograms divided by height in meters squared. For non‐Hispanic Asian participants, elevated BMI was defined as ≥ 27.5 kg/m^2^; for all other participants, the threshold was ≥ 30 kg/m^2^ [4, 8, 9]. BMI and anthropometric cutoffs were based on World Health Organization (WHO) recommendations, which were also adopted by the Excess Adiposity criteria. To align with the Commission's framework, we applied these thresholds in our analysis.

The Excess Adiposity criteria incorporated BMI, body size measurements, and body fat measurement: WC, WHR, WHtR, and, when available, dual‐energy x‐ray absorptiometry (DXA) body fat percentage. Participants met the Excess Adiposity criteria for obesity if they had BMI ≥ 30 kg/m^2^ (≥ 27.5 kg/m^2^ for non‐Hispanic Asian participants) plus ≥ 1 positive body size measurement; BMI ≥ 40 kg/m^2^; ≥ 2 positive body size measurements; or excess adiposity confirmed by DXA.

Body size measurements included WC (≥ 102 cm for non‐Hispanic men, ≥ 88 cm for non‐Hispanic women, ≥ 90 cm for non‐Hispanic Asian men, and ≥ 80 cm for non‐Hispanic Asian women), WHR (> 0.90 for men, > 0.85 for women), WHtR (> 0.50), and DXA body fat percentage (≥ 25% for men, ≥ 35% for women). Body fat percentage data from whole‐body DXA scans were available in NHANES from 2011 to 2016 for ages up to 60. For survey cycles without DXA data, only the anthropometric criteria were applied. Hip circumference measurements were not available until the 2017–2020 cycle. Our primary analyses and interpretations focused on WC and WHtR, which were consistently available across all included survey cycles.

The Excess Adiposity criteria further classify obesity as preclinical (excess adiposity without organ dysfunction) or clinical (organ dysfunction associated with obesity or activity limitations). Organ dysfunction was evaluated across multiple systems, including respiratory, cardiovascular, metabolic, renal, urinary, hepatic, musculoskeletal, and reproductive systems, using biomarkers, physical measurements, and self‐reported measures. Mobility impairments were assessed using self‐reported difficulty performing basic mobility or functional tasks (see online Supporting Information Methods). NHANES does not capture some organ dysfunctions included in the Excess Adiposity criteria (e.g., atrial fibrillation, intracranial hypertension, lymphedema). As a result, only organ dysfunctions that could be reliably identified using self‐reported diagnoses, biomarkers, physical measurements, or specific medication data were included.

### 2.3 Sociodemographic and Clinical Characteristics

Sociodemographic characteristics included age, sex (male or female), race and ethnicity (Hispanic, Non‐Hispanic Asian, non‐Hispanic Black, non‐Hispanic White, or other), family income (< 130%, 130%–349%, or ≥ 350% of the federal poverty level), education (high school or less, some college, or college graduate), and health insurance coverage (uninsured, any health insurance, private, Medicaid, Medicare/Medi‐Gap, or other). Self‐reported health behaviors included smoking status (current smoker, former smoker, or never smoked). Cardiometabolic risk factors included systolic and diastolic blood pressure, fasting glucose, glycated hemoglobin (HbA1c), total cholesterol, high‐density lipoprotein, and low‐density lipoprotein cholesterol levels. Obesity‐associated diseases included diabetes, hypertension, dyslipidemia, cardiovascular disease, metabolic syndrome, metabolic dysfunction‐associated steatotic liver disease (MASLD) with fibrosis, chronic kidney disease, asthma, obstructive sleep apnea, depression, osteoarthritis, urinary incontinence, and female infertility.

### 2.4 Statistical Analysis

We estimated weighted totals and prevalence percentages to generate nationally representative temporal trends. All analyses incorporated the NHANES multistage survey design, including strata, clusters, and combined examination and interview sample weights to produce nationally representative estimates. To improve precision, survey cycles were pooled into 5‐year intervals for earlier years (1999–2004, 2005–2010, 2011–2016). Because NHANES field operations were suspended in 2020 during the COVID‐19 pandemic, the 2017–2020 and 2021–2023 cycles were pooled separately. Temporal trends were assessed using weighted linear regression models that regressed prevalence on the midpoint year of each NHANES survey cycle. A two‐sided *p* value < 0.05 was considered statistically significant. Each estimate was weighted by the inverse of its squared standard error. Means and 95% confidence intervals (CI) were reported. All statistical analyses were conducted in R version 4.2.1. Percentages of missing values for key variables are reported (Table S1). Additional methodological details are provided in the online Supporting Information Methods.

## 3 Results

### 3.1 Prevalence of Obesity by BMI and Excess Adiposity Criteria

**FIGURE 2:**
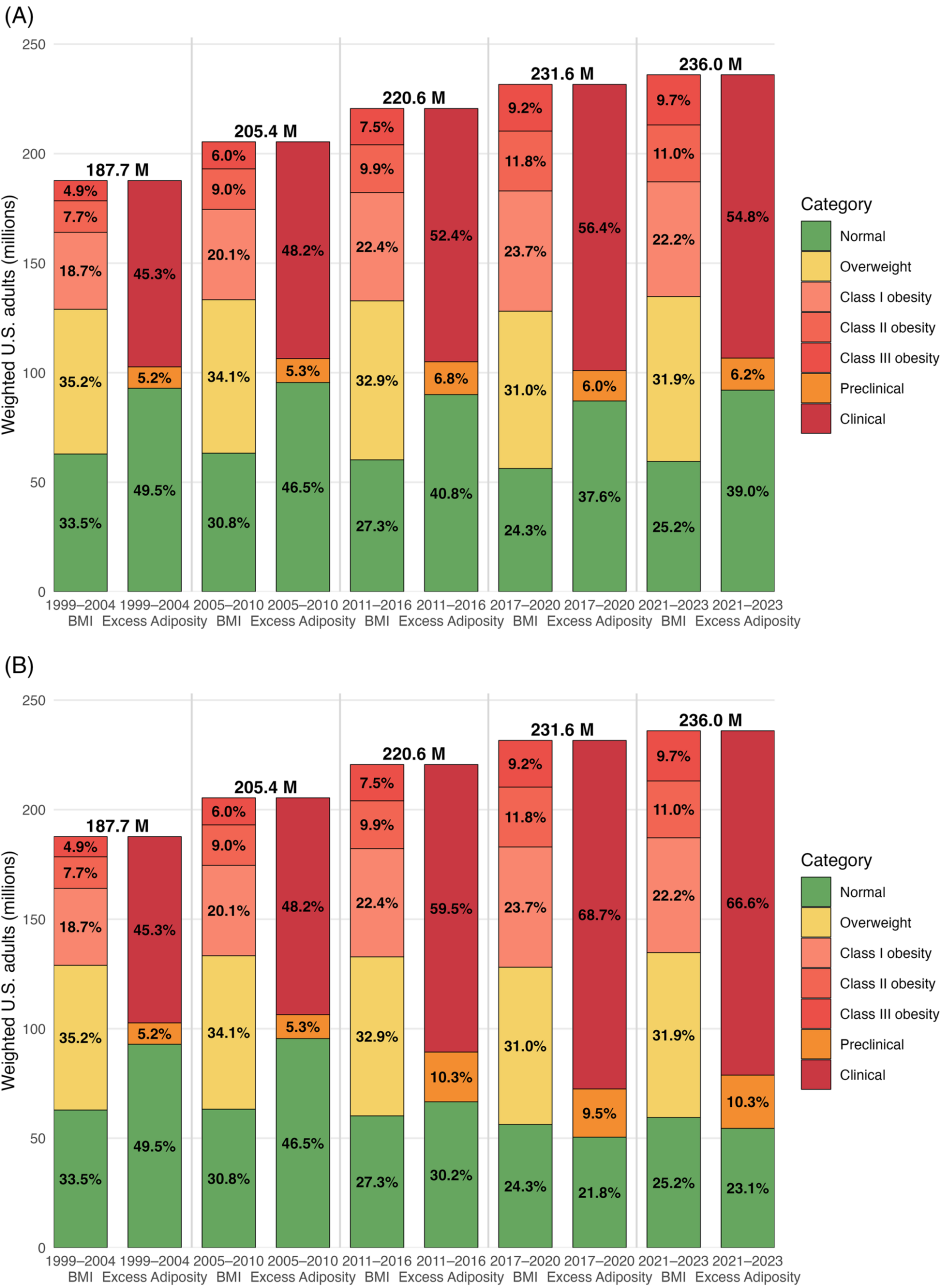
Trends in the prevalence of obesity among US adults: comparison of BMI and Excess Adiposity criteria, NHANES 1999–2023. (A) Obesity excluding body fat measures or WHR due to limited data availability. (B) Obesity including body fat measures 2011–2016 and WHR 2017–2023. BMI categories are defined as normal (BMI < 25), overweight (BMI 25–29.9), Class I (BMI 30–34.9), Class II (BMI 35–39.9), and Class III (BMI ≥ 40). The Excess Adiposity criteria classify individuals as preclinical (excess adiposity without complications) or clinical (excess adiposity with at least one obesity‐associated dysfunction or daily limitations). Weighted totals in millions are displayed above each bar. Dual‐energy x‐ray absorptiometry (DXA) total body fat measures and hip circumference measurements were not available for 1999–2004 and 2005–2010. WHR, waist to hip ratio. [Color figure can be viewed at wileyonlinelibrary.com]

The prevalence of obesity in US adults by both the BMI and the Excess Adiposity criteria increased from 1999 to 2023 (Table 1 and Figure 2). By BMI criteria, the prevalence of obesity increased from 31.3% (58.8 million) in 1999–2004 to 42.9% (101.3 million) in 2021–2023 (*p* for trend < 0.008). Class I obesity increased from 18.7% (35.0 million) to 22.2% (52.4 million; *p* = 0.05), Class II obesity from 7.7% (14.5 million) to 11.0% (25.9 million; *p* = 0.01), and Class III obesity from 4.9% (9.3 million) to 9.7% (22.9 million; *p* < 0.001). During the same period, the proportion of adults with normal BMI declined from 33.5% (62.9 million) to 25.2% (59.4 million; *p* = 0.007).

**TABLE 1:** Trends in population estimates and prevalence of obesity among U.S. adults: comparison of BMI and Excess Adiposity criteria, NHANES 1999–2023.

		1999–2004 (*N* = 12,752)	2005–2010 (*N* = 15,572)	2011–2016 (*N* = 15,708)	2017–2020 (*N* = 8174)	2021–2023 (*N* = 5847)	*p* value
Weighted total, *n* in millions		187.7	205.4	220.6	231.6	236.0	
BMI criteria, *n* in millions (%)	Normal	62.9 (33.5)	63.2 (30.8)	60.2 (27.3)	56.2 (24.3)	59.4 (25.2)	**0.007**
	Overweight	66.1 (35.2)	69.9 (34.1)	72.6 (32.9)	71.7 (31.0)	75.3 (31.9)	**0.03**
	Obesity	58.8 (31.3)	72.3 (35.1)	87.9 (39.8)	103.7 (44.7)	101.3 (42.9)	**0.008**
	Class I	35.0 (18.7)	41.3 (20.1)	49.6 (22.4)	55.0 (23.7)	52.4 (22.2)	0.05
	Class II	14.5 (7.7)	18.6 (9.0)	21.8 (9.9)	27.3 (11.8)	25.9 (11.0)	**0.01**
	Class III	9.3 (4.9)	12.4 (6.0)	16.4 (7.5)	21.4 (9.2)	22.9 (9.7)	**< 0.001**
Excess Adiposity criteria, *n* in millions (%)^a^, ^b^	Normal without body fat or WHR	93.0 (49.5)	95.4 (46.5)	90.1 (40.8)	87.0 (37.6)	92.1 (39.0)	**0.01**
	Obesity without body fat or WHR	94.7 (50.5)	110.0 (53.5)	130.5 (59.2)	144.6 (62.4)	143.9 (61.0)	**0.01**
	Preclinical obesity without body fat or WHR	9.8 (5.2)	11.1 (5.3)	14.9 (6.8)	14.1 (6.0)	14.7 (6.2)	0.37
	Clinical obesity without body fat or WHR	84.9 (45.3)	98.9 (48.2)	115.6 (52.4)	130.5 (56.4)	129.2 (54.8)	**0.03**
	Normal with body fat or WHR	n/a^c^	n/a^c^	66.6 (30.2)	50.5 (21.8)	54.6 (23.1)	0.35
	Obesity with body fat or WHR	n/a^c^	n/a^c^	154.0 (69.8)	181.1 (78.2)	181.4 (76.9)	0.35
	Preclinical obesity with body fat or WHR	n/a^c^	n/a^c^	22.7 (10.3)	22.0 (9.5)	24.3 (10.3)	0.55
	Clinical obesity with body fat or WHR	n/a^c^	n/a^c^	131.3 (59.5)	159.1 (68.7)	157.1 (66.6)	0.54

*Note*: Significant *p* values are indicated in bold. *p* value for time trends.

Abbreviations: n/a, not available; WHR, waist to hip ratio.

^a^ Dual‐energy x‐ray absorptiometry (DXA) total body fat measures were available for adults aged ≤ 60 years during 2011–2016.

^b^ Hip circumference measurements were available for 2017–2023.

^c^ DXA total body fat measures and hip circumference measurements were not available for 1999–2004 and 2005–2010.

When the Excess Adiposity criteria of obesity were applied, the prevalence of obesity was higher at all time points compared to BMI alone (Table 1 and Figure 2). Figure 2A presents estimates using anthropometric measures available across all NHANES cycles to enable temporal comparisons, whereas Figure 2B includes additional measures such as DXA body fat percentage and WHR, which were only collected in selected cycles, to provide more comprehensive estimates. Without body fat or WHR data, the combined prevalence of preclinical and clinical obesity increased from 50.5% (94.7 million) to 61% (143.9 million; *p* = 0.01). When body fat measures (available 2011–2016 for adults ≤ 60 years) and WHR (available 2017–2023) were included, the prevalence was up to 69.8% (154.0 million) in 2011–2016 and 76.9% (181.4 million) in 2021–2023 (*p* = 0.35).

### 3.2 Trends in Preclinical and Clinical Obesity

The prevalence of preclinical obesity was low: 5.2% (9.8 million) in 1999–2004, 5.3% (11.1 million) in 2005–2010, 6.8% (14.9 million) in 2011–2016, 6.0% (14.1 million) in 2017–2020, and 6.2% (14.7 million) in 2021–2023 (*p* = 0.37) (Table 1). When body fat or WHR data were included, the prevalence of preclinical obesity was higher: 10.3% (22.7 million) in 2011–2016, 9.5% (22.0 million) in 2017–2020, and 10.3% (24.3 million) in 2021–2023 (*p* = 0.55). In contrast, clinical obesity increased from 45.3% (84.9 million) in 1999–2004 to 48.2% (98.9 million) in 2005–2010, 52.4% (115.6 million) in 2011–2016, 56.4% (130.5 million) in 2017–2020, and 54.8% (129.2 million) in 2021–2023 (*p* = 0.03) and was higher when including body fat or WHR data: 59.5% (131.3 million) in 2011–2016, 68.7% (159.1 million) in 2017–2020, and 66.6% (157.1 million) in 2021–2023 (*p* = 0.54).

### 3.3 Body Size Measurements and Excess Adiposity Criteria

The increase in obesity defined by Excess Adiposity criteria was due to increases in WC over time and a high prevalence of elevated WHtR (Table 2). WC measurements meeting the Excess Adiposity criteria increased from 49.2% (92.4 million) in 1999–2004 to 59.1% (139.5 million) in 2021–2023 (*p* = 0.02), reflecting a steady upward trend. WHtR showed a similar trend: 76.1% (142.9 million) in 1999–2004 increasing to 81.0% (191.2 million) in 2021–2023 (*p* = 0.01). WHR, which was first reported in 2017–2020, showed that 75.7% (175.2 million) in 2017–2020 and 72.5% (171.1 million) in 2021–2023 met the excess adiposity threshold. Adults meeting two or more positive body size measurements increased from 49.1% (92.3 million) to 75.6% (178.5 million) (*p* = 0.03). Body fat assessment by DXA, available only in 2011–2016, showed that 41.4% (91.4 million) of adults exceeded the Lancet Commission adiposity threshold [9].

**TABLE 2:** Trend in population estimates and prevalence of excess adiposity in U.S. adults according to Excess Adiposity Criteria, NHANES 1999–2023.

		1999–2004 (*N* = 12,752)	2005–2010 (*N* = 15,572)	2011–2016 (*N* = 15,708)	2017–2020 (*N* = 8,174)	2021–2023 (*N* = 5,847)	*p* value
Body size measurement confirming excess adiposity, *n* in millions (%)	Waist circumference	92.4 (49.2)	106.9 (52.1)	126.8 (57.5)	140.7 (60.7)	139.5 (59.1)	**0.02**
	Waist to height ratio	142.9 (76.1)	158.2 (77.0)	176.3 (79.9)	188.6 (81.4)	191.2 (81.0)	**0.01**
	Waist to hip ratio	n/a^a^	n/a^a^	n/a^a^	175.2 (75.7)	171.1 (72.5)	n/a
Excess Adiposity criteria, *n* in millions (%)	Two or more positive body size measurements	92.3 (49.1)	106.8 (52.0)	126.5 (57.3)	179.0 (77.3)	178.5 (75.6)	**0.03**
	Body fat measurement	n/a^b^	n/a^b^	91.4 (41.4)	n/a^b^	n/a^b^	n/a^b^
	BMI ≥ 30 kg/m^2^ with at least one positive body size measure	57.0 (30.3)	68.7 (33.5)	82.3 (37.3)	96.9 (41.8)	94.3 (40.0)	**0.007**
	BMI ≥ 40kg/m^2^	9.3 (4.9)	12.4 (6.0)	16.4 (7.5)	21.4 (9.2)	22.9 (9.7)	**< 0.001**

*Note*: Significant *p* values are indicated in bold. *p* value for time trends.

Abbreviation: n/a, not available.

^a^ Hip circumference measurements were available for 2017–2023.

^b^ Dual‐energy x‐ray absorptiometry (DXA) total body fat measures were available for adults aged ≤ 60 years during 2011–2016.

The proportion with BMI ≥ 30 kg/m^2^ and at least one positive body size measure increased from 30.3% (57 million) to 40.0% (94.3 million) from 1999 to 2023 (*p* = 0.007). The proportion of adults with BMI ≥ 40 kg/m^2^ nearly doubled from 4.9% (9.3 million) to 9.7% (22.9 million) over the same time (*p* < 0.001).

### 3.4 Obesity Among Adults With Normal or Overweight BMI

The proportion of US adults with normal or overweight BMI who were considered as having obesity (45 million) by Excess Adiposity criteria remained stable over time (*p* = 0.77 for normal BMI; *p* = 0.29 for overweight BMI) (Table S2A). Among adults with normal BMI, the individuals who had obesity by Excess Obesity criteria remained stable from 2.6% (4.8 million) to 2.6% (6.2 million) (*p* = 0.77). Preclinical obesity (*p* = 0.52) and clinical obesity did not change significantly (*p* = 0.59).

Among adults with overweight BMI, the prevalence of obesity by Excess Adiposity criteria remained stable from 17.3% (32.5 million) to 16.7% (39.5 million) (*p* = 0.29). Preclinical obesity did not change significantly (*p* = 0.14), while clinical obesity changed from 15.2% (28.5 million) to 14.6% (34.5 million) (*p* = 0.03).

Among individuals with obesity by BMI criteria, 30.6% (57.4 million) to 40.8% (96.2 million) met the Excess Adiposity criteria across all survey periods (*p* = 0.007). Within this group, preclinical obesity was low (e.g., 3.6% [8.6 million] in 2021–2023; *p* = 0.12), whereas clinical obesity increased from 27.8% (52.2 million) to 37.1% (87.6 million) (*p* = 0.006). In Class I obesity, preclinical obesity was low (*p* = 0.26), while clinical obesity increased from 16.4% (30.8 million) to 18.9% (44.6 million) (*p* = 0.02). In Class II, preclinical obesity increased from 0.6% (1.2 million) to 0.7% (1.7 million) (*p* = 0.03), whereas clinical obesity was higher at 6.8% (12.7 million) to 9.9% (23.3 million) (*p* = 0.008). In Class III, all individuals met the Excess Adiposity criteria for obesity. In this group, preclinical obesity increased from 0.3% (0.5 million) to 0.6% (1.4 million) (*p* = 0.05), and clinical obesity increased from 4.7% (8.7 million) to 9.1% (21.5 million) (*p* < 0.001).

### 3.5 Trends in Sociodemographic Characteristics of Adults With Obesity

From 1999 to 2023, US adults with obesity defined by either BMI or Excess Adiposity criteria became older (Table 3 and Table S3). The mean age increased by both BMI criteria (*p* = 0.02) and Excess Adiposity criteria (*p* = 0.005). The prevalence of obesity based on BMI and Excess Adiposity criteria was higher among females than males from 1999 to 2016, but the rates became similar in more recent years. For example, among females, obesity prevalence increased from 33.9% (95% CI 32.5%–35.3%) to 42.9% (40.8%–45.0%) by BMI criteria (*p* = 0.01) and from 59.3% (57.8%–60.8%) to 69.7% (67.6%–71.7%) by Excess Adiposity criteria (*p* = 0.03).

**TABLE 3:** Sociodemographic, anthropometric, and clinical characteristics of adult NHANES participants by BMI and Excess Adiposity Criteria for obesity, 1999–2023.

Obesity not including body fat measures or WHR												
	1999–2004		2005–2010		2011–2016		2017–2020		2021–2023		*p* value for BMI criteria	*p* value for Excess Adiposity criteria
	Obesity by BMI criteria	Obesity by Excess Adiposity criteria	Obesity by BMI criteria	Obesity by Excess Adiposity criteria	Obesity by BMI criteria	Obesity by Excess Adiposity criteria	Obesity by BMI criteria	Obesity by Excess Adiposity criteria	Obesity by BMI criteria	Obesity by Excess Adiposity criteria		
Age, % (95% CI)												
Mean (95% CI)	47.3 (46.8–47.8)	49.7 (49.3–50.2)	48.0 (47.6–48.5)	50.1 (49.8–50.5)	48.7 (48.0–49.3)	50.3 (49.8–50.9)	48.8 (47.8–49.8)	50.9 (49.9–51.8)	49.8 (48.8–50.7)	51.6 (50.7–52.6)	**0.02**	**0.005**
20–44 years	29 (27–30)	40.7 (39.2–42.3)	32.4 (31.1–33.7)	43.8 (42.4–45.3)	42.1 (40.2–43.9)	38.0 (36.4–39.6)	42.6 (39.8–45.4)	37.4 (34.8–40.1)	40.3 (37.8–42.7)	36.9 (34.6–39.1)	0.06	0.20
45–64 years	35.8 (34.0–37.6)	58.8 (56.9–60.7)	39.4 (37.8–41.1)	60.7 (59.0–62.4)	39.6 (38.1–41.1)	40.2 (38.8–41.6)	37.0 (34.3–39.7)	38.6 (36.4–40.8)	38.6 (37.1–40.2)	37.5 (35.4–39.5)	0.56	0.07
≥ 65 years	29.8 (27.9–31.7)	62.7 (60.8–64.7)	33.9 (32.1–35.7)	65.0 (63.2–66.8)	18.3 (16.9–19.7)	21.7 (20.5–23.0)	20.4 (17.6–23.2)	23.9 (21.3–26.6)	21.1 (19.0–23.2)	25.6 (22.9–28.3)	0.22	0.12
Sex, % (95% CI)												
Female	33.9 (32.5–35.3)	59.3 (57.8–60.8)	36.6 (35.2–37.9)	61.5 (60.1–62.8)	40.8 (39.4–42.3)	68.2 (66.8–69.5)	43.9 (41.7–46.1)	70.4 (68.3–72.5)	42.9 (40.8–45.0)	69.7 (67.6–71.7)	**0.01**	**0.03**
Male	28.6 (27.2–30.0)	41.3 (39.8–42.8)	33.8 (32.5–35.1)	45.3 (43.9–46.7)	36.7 (35.3–38.2)	49.7 (48.2–51.2)	43.0 (40.6–45.4)	54.1 (51.6–56.5)	40.7 (38.3–43.0)	52.1 (49.7–54.4)	**0.01**	**0.01**
Race/ethnicity, % (95% CI)^**a**^												
Non–Hispanic White	30.5 (29.2–31.7)	51.2 (49.9–52.6)	34.1 (32.9–35.3)	54.4 (53.2–55.7)	36.9 (35.5–38.3)	58.8 (57.4–60.3)	42.1 (39.7–44.5)	62.5 (60.1–64.9)	40.2 (38.3–42.2)	60.9 (58.9–62.9)	**0.01**	**0.01**
Non–Hispanic Black	41.4 (39.4–43.5)	54.6 (52.5–56.7)	47.0 (45.2–48.9)	58.3 (56.5–60.2)	48.5 (46.7–50.3)	61.0 (59.3–62.7)	50.8 (48.3–53.2)	63.1 (60.6–65.5)	52.1 (47.5–56.6)	64.2 (59.8–68.6)	**0.02**	**0.003**
Hispanic	31.3 (29.1–33.5)	47.6 (45.2–50.0)	36.7 (35.0–38.3)	52.8 (51.0–54.6)	44.3 (42.5–46.0)	60.0 (58.3–61.8)	45.7 (42.9–48.4)	60.0 (57.3–62.8)	44.3 (40.4–48.2)	59.1 (55.2–63.1)	**0.03**	0.05
Non–Hispanic Asian	n/a	n/a	n/a	n/a	24.3 (22.3–26.4)	56.7 (54.4–59.0)	33.0 (29.8–36.3)	64.7 (61.4–68.0)	28.6 (22.2–35.0)	61.9 (55.1–68.7)	0.42	0.37
Other	20.4 (16.3–24.6)	37.1 (32.0–42.1)	22.1 (18.6–25.6)	35.7 (31.7–39.8)	43.7 (37.9–49.6)	59.2 (53.6–64.9)	50.6 (43.4–57.9)	65.6 (58.9–72.3)	45.3 (39.0–51.5)	60.5 (54.5–66.6)	**0.04**	0.06
Education level, % (95% CI)												
High school or less	33.6 (32.2–35.0)	53.8 (52.3–55.3)	37.9 (36.5–39.2)	56.7 (55.3–58.1)	41.8 (40.2–43.3)	61.8 (60.3–63.3)	45.9 (43.3–48.5)	63.8 (61.4–66.3)	45.7 (43.0–48.4)	64.6 (62.0–67.2)	**0.004**	**0.007**
Some college	32.7 (30.8–34.6)	51.9 (49.8–53.9)	38.8 (37.0–40.5)	56.5 (54.7–58.3)	43.4 (41.5–45.2)	63.1 (61.3–64.9)	47.8 (45.1–50.6)	66.0 (63.3–68.6)	46.9 (44.1–49.8)	64.8 (62.0–67.6)	**0.01**	**0.02**
College graduate	25.1 (23.1–27.1)	42.2 (39.9–44.5)	26.7 (24.9–28.5)	45.1 (43.1–47.2)	30.7 (28.8–32.6)	52.0 (49.9–54.0)	36.4 (33.3–39.6)	57.5 (54.1–60.8)	33.3 (30.8–35.8)	53.9 (51.2–56.5)	**0.03**	**0.03**
Family poverty income (FPL) ratio, % (95% CI)												
Less than 130% of FPL	33.8 (31.7–35.8)	53.0 (50.8–55.2)	37.9 (36.2–39.6)	55.7 (53.9–57.5)	41.0 (39.3–42.7)	59.2 (57.4–60.9)	45.7 (42.6–48.8)	62.6 (59.6–65.7)	45.8 (42.0–49.5)	63.0 (59.4–66.7)	**0.003**	**0.003**
130%–349% of FPL	33.1 (31.4–34.8)	52.6 (50.8–54.5)	37.1 (35.5–38.7)	55.7 (54.1–57.4)	41.7 (39.9–43.5)	61.5 (59.8–63.3)	48.0 (45.3–50.7)	65.5 (62.9–68.1)	44.4 (41.7–47.1)	63.1 (60.3–65.8)	**0.02**	**0.02**
≥ 350% of FPL	28.4 (26.8–30.1)	47.3 (45.5–49.1)	32.9 (31.3–34.5)	51.4 (49.7–53.1)	35.4 (33.5–37.2)	57.6 (55.7–59.5)	40.7 (37.7–43.6)	61.5 (58.5–64.4)	38.0 (35.5–40.6)	58.8 (56.2–61.4)	**0.02**	**0.03**
Insurance status, % (95% CI)												
No insurance	28.9 (26.7–31.1)	44.0 (41.6–46.5)	31.9 (30.0–33.7)	46.0 (44.0–48.0)	38.9 (36.8–41.0)	54.6 (52.5–56.8)	39.2 (35.6–42.8)	52.0 (48.2–55.9)	43.1 (37.7–48.4)	54.7 (49.3–60.0)	**0.01**	0.07
Any insurance	31.8 (30.7–32.9)	52.0 (50.8–53.2)	36.0 (35.0–37.1)	55.4 (54.3–56.5)	38.8 (37.7–40.0)	60.1 (58.9–61.3)	44.1 (42.3–45.9)	64.0 (62.2–65.8)	41.6 (40.0–43.2)	61.6 (60.0–63.3)	**0.02**	**0.024**
Medicaid	40.7 (36.4–45.0)	63.9 (59.8–68.1)	47.1 (43.5–50.6)	64.1 (60.7–67.5)	48.0 (45.0–51.0)	63.6 (60.8–66.5)	48.6 (44.6–52.7)	66.2 (62.5–69.9)	48.3 (43.8–52.7)	64.0 (59.6–68.5)	0.13	0.53
Medicare/Medi–Gap	31.9 (29.8–34.0)	63.5 (61.4–65.6)	35.3 (33.4–37.2)	66.0 (64.1–67.9)	38.5 (36.1–40.9)	69.2 (67.0–71.4)	42.6 (39.4–45.8)	70.7 (67.6–73.8)	40.3 (37.7–42.8)	69.6 (67.2–72.0)	**0.02**	**0.03**
Private	31.0 (29.6–32.4)	47.7 (46.3–49.2)	34.9 (33.5–36.2)	51.3 (49.9–52.8)	37.5 (35.9–39.0)	56.7 (55.1–58.3)	43.4 (40.8–46.1)	60.8 (58.1–63.4)	40.1 (37.6–42.6)	57.8 (55.3–60.2)	**0.02**	**0.03**
Other	35.9 (29.0–42.8)	60.0 (52.9–67.1)	41.3 (37.1–45.6)	60.0 (55.7–64.3)	40.2 (36.3–44.1)	59.6 (55.7–63.5)	46.4 (40.4–52.3)	64.3 (58.4–70.2)	44.2 (38.4–50.0)	62.0 (56.2–67.9)	0.11	0.25
Smoking status, % (95% CI)^**b**^												
Never smoker	32.1 (30.7–33.5)	50.6 (49.0–52.1)	36.3 (35.0–37.6)	53.4 (52.0–54.7)	38.1 (36.8–39.4)	58.1 (56.7–59.5)	43.4 (41.3–45.6)	62.6 (60.5–64.7)	40.5 (38.5–42.5)	60.1 (58.1–62.2)	**0.03**	**0.02**
Former smoker	33.7 (31.7–35.7)	56.3 (54.2–58.4)	37.8 (35.9–39.7)	59.7 (57.8–61.6)	42.6 (40.4–44.8)	64.9 (62.8–67.0)	47.5 (44.0–50.9)	65.8 (62.3–69.3)	46.4 (43.3–49.5)	65.0 (62.0–67.9)	**0.007**	**0.04**
Current smoker	27.3 (25.4–29.3)	44.3 (42.1–46.4)	29.6 (27.8–31.5)	47.1 (45.0–49.1)	36.3 (34.0–38.5)	54.9 (52.6–57.2)	37.2 (33.5–40.9)	56.7 (52.9–60.5)	39.9 (35.8–43.9)	58.5 (54.4–62.6)	**0.01**	**0.01**
Anthropometric measures												
Mean weight, kg (95% CI)	100.9 (100.2–101.6)	91.7 (91.1–92.3)	101.8 (101.2–102.4)	92.9 (92.4–93.5)	101.4 (100.7–102.0)	92.2 (91.6–92.8)	102.6 (101.6–103.6)	93.9 (92.9–94.8)	102.5 (101.5–103.5)	89.4 (88.6–90.2)	0.09	0.53
Mean BMI, kg/m^2^ (95% CI)	35.5 (35.3–35.7)	32.3 (32.1–32.5)	35.7 (35.5–35.9)	32.7 (32.5–32.9)	35.9 (35.7–36.1)	32.7 (32.5–32.9)	36.3 (36.0–36.6)	33.4 (33.1–33.6)	36.5 (36.2–36.8)	31.7 (31.5–32.0)	**0.001**	0.92
Mean waist circumference, cm (95% CI)	112.6 (112.2–113.1)	107.1 (106.8–107.5)	113.9 (113.5–114.3)	108.2 (107.9–108.6)	114.7 (114.3–115.2)	108.6 (108.2–109.0)	115.2 (114.5–115.8)	109.6 (109.0–110.2)	115.4 (114.7–116.0)	106.0 (105.4–106.5)	**0.01**	0.93
Cardiometabolic risk factors												
Mean systolic blood pressure, mmHg (95% CI)	127.1 (126.4–127.8)	127.5 (126.9–128.1)	124.7 (124.2–125.3)	124.5 (124.0–124.9)	125.6 (125.1–126.2)	124.7 (124.2–125.2)	123.2 (122.3–124.0)	123.4 (122.6–124.1)	122.6 (121.8–123.4)	122.8 (122.2–123.4)	0.07	0.05
Mean diastolic blood pressure, mmHg (95% CI)	74.1 (73.6–74.6)	73.2 (72.8–73.6)	72.0 (71.6–72.4)	71.2 (70.8–71.5)	72.1 (71.7–72.5)	71.5 (71.1–71.8)	76.8 (76.3–77.4)	75.6 (75.1–76.1)	77.9 (77.4–78.5)	76.1 (75.7–76.5)	0.21	0.20
Mean glucose, mg/dL (95% CI)	108.1 (106.2–110.1)	106.9 (105.3–108.5)	112.9 (111.2–114.6)	110.4 (109.0–111.7)	115.4 (113.5–117.4)	112.0 (110.5–113.4)	116.0 (113.5–118.6)	113.6 (111.6–115.7)	115.7 (113.0–118.5)	112.4 (110.5–114.3)	0.05	0.06
Mean HbA1c, % (95% CI)	5.7 (5.7–5.8)	5.7 (5.6–5.7)	5.8 (5.8–5.8)	5.7 (5.7–5.8)	5.9 (5.9–5.9)	5.8 (5.8–5.8)	5.9 (5.8–5.9)	5.8 (5.8–5.9)	6.0 (5.9–6.0)	5.8 (5.8–5.9)	**0.006**	0.06
Mean cholesterol, mg/dL (95% CI)	205.7 (204.0–207.3)	208.1 (206.8–209.4)	198.2 (196.8–199.5)	200.8 (199.7–202.0)	193.4 (192.0–194.9)	195.3 (194.1–196.5)	187.9 (185.9–190.0)	189.7 (188.0–191.4)	189.0 (186.9–191.1)	191.0 (189.4–192.5)	**0.01**	**0.01**
Mean high‐density lipoprotein, mg/dL (95% CI)	46.4 (45.9–46.9)	49.0 (48.6–49.5)	47.0 (46.6–47.5)	49.7 (49.3–50.1)	48.0 (47.5–48.5)	50.8 (50.4–51.3)	48.4 (47.8–49.1)	51.1 (50.5–51.7)	49.3 (48.7–49.9)	51.8 (51.2–52.3)	**0.001**	**0.002**
Mean low‐density lipoprotein, mg/dL (95% CI)	124.4 (122.4–126.5)	124.9 (123.3–126.5)	117.0 (115.3–118.7)	118.8 (117.4–120.2)	115.1 (113.4–116.9)	116.0 (114.5–117.4)	110.5 (107.9–113.0)	111.5 (109.3–113.6)	n/a	n/a	**0.04**	**0.02**
Chronic disease, % (95% CI)												
Diabetes^c^	53.9 (50.9–56.9)	75.9 (73.4–78.5)	61.3 (58.9–63.6)	78.7 (76.7–80.7)	62.0 (59.6–64.4)	80.2 (78.2–82.2)	64.3 (60.8–67.7)	81.3 (78.5–84.1)	62.9 (59.6–66.2)	81.6 (79.0–84.3)	0.12	**0.02**
Hypertension^d^	39.5 (38.0–40.9)	62.1 (60.7–63.6)	45.7 (44.3–47.1)	66.9 (65.5–68.2)	48.9 (47.4–50.4)	70.5 (69.2–71.9)	54.0 (51.7–56.2)	74.5 (72.5–76.4)	52.9 (50.9–55.0)	73.5 (71.7–75.4)	**0.01**	**0.01**
Dyslipidemia^e^	41.1 (39.6–42.7)	63.5 (61.9–65.0)	46.7 (45.3–48.1)	68.0 (66.7–69.3)	50.9 (49.3–52.4)	72.9 (71.6–74.3)	53.9 (51.6–56.3)	74.7 (72.6–76.7)	n/a	n/a	**0.01**	**0.02**
Cardiovascular disease^f^	40.0 (36.8–43.1)	65.6 (62.5–68.7)	45.0 (42.2–47.8)	70.8 (68.2–73.4)	49.3 (46.1–52.5)	67.5 (64.5–70.5)	51.6 (46.9–56.3)	71.6 (67.3–75.8)	53.6 (49.2–58.1)	73.5 (69.6–77.4)	**0.004**	0.17
Pulmonary hypertension^b^	33.0 (0.0–68.4)	70.2 (35.7–100.0)	24.4 (5.3–43.5)	65.5 (44.4–86.6)	32.7 (12.0–53.5)	43.7 (21.8–65.7)	49.7 (25.1–74.3)	66.5 (43.3–89.7)	n/a	n/a	**0.22**	0.74
Metabolic syndrome^g^	58.2 (56.2–60.3)	89.2 (87.9–90.4)	64.0 (62.2–65.8)	92.0 (91.0–93.0)	67.4 (65.5–69.2)	93.3 (92.3–94.3)	70.9 (68.1–73.7)	94.8 (93.5–96.2)	67.6 (64.8–70.4)	93.3 (91.9–94.8)	0.06	0.08
MASLD with fibrosis^h^	n/a	n/a	n/a	n/a	n/a	n/a	79.4 (73.9–84.8)	88.7 (84.4–92.9)	71.2 (65.8–76.6)	84.9 (80.6–89.2)	n/a	n/a
Chronic kidney disease^i^	34.6 (30.8–38.5)	65.5 (61.6–69.3)	42.0 (38.7–45.3)	69.6 (66.5–72.7)	41.8 (38.0–45.5)	65.3 (61.7–69.0)	49.4 (43.9–55.0)	76.9 (72.2–81.6)	n/a	n/a	0.09	0.40
Asthma^j^	42.1 (37.4–46.9)	59.3 (54.5–64.0)	48.9 (45.4–52.4)	65.9 (62.6–69.3)	48.6 (45.1–52.1)	66.8 (63.5–70.1)	54.0 (48.8–59.2)	71.5 (66.8–76.2)	50.4 (45.7–55.2)	68.1 (63.7–72.6)	0.14	0.11
Sleep apnea^j^	n/a	n/a	73.5 (69.5–77.5)	82.5 (79.0–85.9)	73.4 (67.9–78.9)	81.5 (76.7–86.3)	72.9 (68.6–77.2)	83.2 (79.6–86.8)	n/a	n/a	0.20	0.68
Depression^k^	34.2 (25.0–43.4)	41.1 (31.5–50.6)	44.2 (40.8–47.6)	65.5 (62.2–68.7)	50.5 (46.9–54.2)	69.0 (65.7–72.4)	51.6 (46.1–57.1)	68.5 (63.4–73.6)	47.8 (43.1–52.5)	64.9 (60.5–69.4)	0.25	0.58
Osteoarthritis^b^	40.5 (36.9–44.1)	67.8 (64.4–71.2)	43.1 (39.8–46.3)	69.7 (66.7–72.8)	49.6 (46.5–52.7)	73.0 (70.3–75.7)	50.0 (45.4–54.5)	75.2 (71.3–79.1)	53.2 (49.6–56.9)	76.2 (73.1–79.3)	**0.01**	**0.02**
Urinary incontinence^b^	n/a	n/a	43.6 (41.7–45.6)	69.3 (67.5–71.1)	47.4 (45.3–49.4)	71.9 (70.1–73.8)	52.6 (49.8–55.4)	76.1 (73.7–78.4)	47.2 (44.7–49.7)	71.5 (69.3–73.8)	0.36	0.43
Female infertility^b^	n/a	n/a	n/a	n/a	53.7 (48.0–59.5)	74.1 (69.0–79.1)	49.7 (42.0–57.5)	72.7 (65.7–79.6)	n/a	n/a	n/a	n/a

*Note*: Significant *p* values are indicated in bold. *p* value for time trends.

Abbreviations: n/a, not available; MASLD, metabolic dysfunction‐associated steatotic liver disease; WHR, waist to hip ratio.

^a^ Non‐Hispanic Asian participants were not included in 1999–2004 because this group was not sampled prior to 2011–2012 cycle.

^b^ Self‐reported.

^c^ Diabetes is defined as a self‐reported diagnosis, the use of diabetes medication, fasting plasma glucose (FPG) ≥ 125 mg/dL, or HbA1c ≥ 6.5%.

^d^ Hypertension is defined as a self‐reported diagnosis, current treatment for hypertension, or average systolic blood pressure ≥ 130 mmHg or diastolic blood pressure ≥ 80 mmHg.

^e^ Dyslipidemia is defined as a self‐reported diagnosis, current treatment for hyperlipidemia, low‐density lipoprotein cholesterol (LDL‐C) ≥ 160 mg/dL, triglycerides ≥ 150 mg/dL, or high‐density lipoprotein cholesterol (HDL‐C) < 40 mg/dL in men or < 50 mg/dL in women.

^f^ Cardiovascular disease (CVD) history is defined as self‐reported coronary heart disease, congestive heart failure, myocardial infarction, angina, or stroke, or prescription medication use for angina.

^g^ Metabolic syndrome is defined as meeting ≥ 3 of the following: abdominal obesity (waist circumference ≥ 88 cm for women or ≥ 102 cm for men), triglycerides ≥ 150 mg/dL, HDL‐C < 40 mg/dL in men or < 50 mg/dL in women, blood pressure ≥ 130/85 mmHg or antihypertensive use, and fasting glucose ≥ 100 mg/dL or HbA1c ≥ 5.7%.

^h^ MASLD with fibrosis is defined as the presence of hepatic steatosis, indicated by a controlled attenuation parameter (CAP) ≥ 263 dB/m with at least one metabolic risk factor and liver stiffness ≥ 8.6 kPa, excluding participants with excess alcohol intake.[15, 16, 17, 18].

^i^ Chronic kidney disease (CKD) is defined as estimated glomerular filtration rate (eGFR) < 60 mL/min/1.73 m^2^, calculated using the CKD‐EPI 2009 equation.

^j^ Obstructive sleep apnea (OSA) is defined using the STOP‐BANG score (snoring, tiredness, observed apneas, hypertension, BMI ≥ 35, age > 50, and male sex), with a score ≥ 5 indicating OSA. Data are available for 2005–2006, 2007–2008, 2015–2016, and 2017–2020.

^k^ Depression is defined by meeting DSM‐IV diagnostic criteria or a positive result on the PHQ‐9 questionnaire.

The prevalence of obesity was persistently higher among non‐Hispanic Black individuals. Among non‐Hispanic Black adults, the prevalence increased from 41.4% (39.4%–43.5%) in 1999–2004 to 52.1% (47.5%–56.6%) in 2021–2023 by BMI criteria (*p* = 0.02) and from 54.6% (52.5%–56.7%) to 64.2% (59.8%–68.6%) by Excess Adiposity criteria (*p* = 0.003). Among Hispanic adults, the prevalence of obesity increased with nearly half meeting BMI criteria (*p* = 0.03) and over half meeting the Excess Adiposity definition (*p* = 0.05) in 2021–2023. A similar trend was also observed among non‐Hispanic White adults (*p* = 0.01 for both criteria). Among non‐Hispanic Asian adults, the prevalence of obesity increased from 24.3% (22.3%–26.4%) in 2011–2016 to 28.6% (22.2%–35.0%) in 2021–2023 by BMI criteria (*p* = 0.42) and 56.7% (54.4%–59.0%) to 61.9% (55.1%–68.7%) by Excess Adiposity criteria (*p* = 0.37).

Across education levels, adults with a high school education (*p* = 0.004 for BMI criteria; *p* = 0.007 for Excess Adiposity Criteria) or less and those with some college (*p* = 0.01 for BMI criteria; *p* = 0.02 for Excess Adiposity Criteria) consistently had a higher prevalence of obesity by both BMI and Excess Adiposity criteria across all survey periods. Across income levels, adults with lower family poverty income ratios consistently had a higher prevalence of obesity by both BMI and Excess Adiposity criteria (*p* = 0.003 for both criteria). By insurance type, Medicare beneficiaries had the highest prevalence of obesity by Excess Adiposity criteria (*p* = 0.03). By smoking status, former smokers consistently had the highest obesity prevalence by both criteria (*p* = 0.007 for BMI criteria; *p* = 0.04 for Excess Adiposity criteria).

### 3.6 Trends in Anthropometric and Clinical Characteristics of Adults With Obesity

From 1999 to 2023, adults with obesity defined by BMI criteria showed increases in both mean BMI and WC, from 35.5 kg/m^2^ (35.3–35.7) to 36.5 kg/m^2^ (36.2–36.8) (*p* = 0.001) and from 112.6 cm (112.2–113.1) to 115.4 cm (114.7–116.0) (*p* = 0.01), respectively (Table 3). Among adults classified with obesity by Excess Adiposity criteria, mean BMI and WC were relatively unchanged.

Cardiometabolic risk profiles showed mixed patterns from 1999 to 2023. Mean systolic blood pressure decreased, whereas diastolic blood pressure increased among adults with obesity by both BMI and Excess Adiposity criteria, though not statistically significantly. Mean HbA1c increased from 5.7% (5.7%–5.8%) to 6.0% (5.9%–6.0%) by BMI criteria (*p* = 0.006) but did not change significantly for Excess Adiposity criteria (*p* = 0.06). In contrast, lipid profiles improved during the same period. For example, total cholesterol decreased from 205.7 mg/dL (204.0–207.3) to 189.0 mg/dL (186.9–191.1) by BMI criteria (*p* = 0.01) and from 208.1 mg/dL (206.8–209.4) to 191.0 (189.4–192.5) mg/dL by Excess Adiposity criteria (*p* = 0.01).

The prevalence of obesity increased in hypertension (*p* = 0.01 for both criteria), dyslipidemia (*p* = 0.01 for BMI criteria; *p* = 0.02 for Excess Adiposity criteria), metabolic syndrome (*p* = 0.06 for BMI criteria; *p* = 0.08 for Excess Adiposity criteria) from 1999 to 2023 by both criteria. Among adults with diabetes, obesity prevalence increased from 75.9% (73.4%–78.5%) to 81.6% (79.0%–84.3%) by Excess Adiposity criteria (*p* = 0.02). The prevalence of obesity by BMI criteria increased among adults with cardiovascular disease (*p* = 0.004) and osteoarthritis (*p* = 0.01). The prevalence of obesity with pulmonary hypertension, MASLD with fibrosis, chronic kidney disease, asthma, sleep apnea, depression, and urinary incontinence was higher over time, although the change was not statistically significant.

## 4 Discussion

This study found that expanding the definition of obesity using the Excess Adiposity criteria, compared with BMI alone, increased the prevalence of obesity to over 60% of US adults. Furthermore, about 45 million adults with normal or overweight BMI were newly classified as having obesity under the Excess Adiposity criteria. The prevalence of clinical obesity increased significantly over time, whereas preclinical obesity remained unchanged. Importantly, the body size measurements led to a large increase in the percentage of US adults meeting the Excess Adiposity criteria. Obesity prevalence was particularly high among those with hypertension, dyslipidemia, and metabolic syndrome.

Our study found that the Excess Adiposity criteria broaden the population of adults characterized as having obesity well beyond BMI alone. An additional 6.2 million adults with normal BMI and 39.5 million with overweight BMI were considered as having obesity under the Excess Adiposity criteria. There are major implications to this large increase in the number of adults who are considered as having obesity. Because the Excess Adiposity criteria identify nearly three in five US adults as having obesity, health care systems and insurers will face a much larger population potentially eligible for pharmacologic or surgical treatment. The new criteria could expand treatment eligibility, increase demand for health care resources, and challenge current reimbursement and prevention strategies in the era of costly but effective pharmacotherapy [19, 20]. This expansion could raise concerns about the cost‐effectiveness and budget impact of providing coverage for high‐cost medications and procedures, particularly if payers such as Medicare extend coverage for obesity medications [21, 22]. Policy makers and clinicians will need to prioritize individuals at highest risk and consider frameworks to stage obesity severity to guide equitable and sustainable treatment allocation. One such framework is the Edmonton Obesity Staging System (EOSS), which has been proposed to inform coverage decisions and determine which patients are most likely to benefit from intensive therapies [23, 24, 25, 26].

Additionally, while this broader classification may improve identification of individuals at risk for obesity‐associated complications, it also raises concerns regarding potential overclassification, particularly when diagnostic sensitivity varies across anthropometric measures and population subgroups. These considerations underscore the importance of ensuring that broader definitions of obesity remain clinically meaningful and do not overwhelm health care systems with increased demand.

The small proportion of adults classified with preclinical obesity suggests that most individuals with excess adiposity already exhibit organ dysfunction and/or limitations in daily activities by the time obesity is diagnosed. This finding may indicate a narrow window for early intervention before the development of obesity‐associated organ dysfunction and impairment. Moreover, the high prevalence of obesity by the Excess Adiposity criteria adds another layer of complexity to an important public health challenge. Meaningful progress will require addressing both individual and structural determinants of obesity, recognizing that it affects a large proportion of the population. Efforts should focus on improving access to healthy foods and opportunities for physical activity, reducing socioeconomic inequities, and strengthening clinical strategies to reduce the overall burden of obesity [27, 28, 29].

In our analysis, a higher proportion of adults with cardiometabolic diseases were categorized as having obesity by the Excess Adiposity criteria than the BMI criteria. This pattern was consistent particularly in hypertension, dyslipidemia, and metabolic syndrome. These findings suggest that the Excess Adiposity criteria identify a broader population with obesity‐associated disease burden. Thus, using the Excess Adiposity criteria may allow for earlier detection and intervention. However, a major challenge is that clinical obesity defined by the Excess Adiposity criteria can be highly heterogeneous in the severity of organ dysfunction or activity limitations. Across studies, the application of this definition has been inconsistent, reflecting the lack of standardized diagnostic approaches [11, 12, 13, 30]. There is a need for greater clarity and consensus on the diagnostic criteria used to define clinical obesity in both research and clinical settings, particularly regarding the organ dysfunction and activity limitations attributable to excess adiposity. In addition, the absence of validated, practical tools to evaluate and diagnose organ dysfunction and daily activity limitation associated with obesity may continue to hinder the integration of the Excess Adiposity criteria into routine clinical care and clinical trials. Development of obesity severity staging frameworks may help address this gap by supporting individualized treatment decisions and facilitating longitudinal monitoring of disease progression [31, 32, 33, 34].

Incorporating multiple anthropometric measures within this new framework can improve identifying high‐risk individuals who would otherwise be missed by the BMI criteria alone [35]. However, there are potential limitations. For example, the current WHtR cutoff of 0.5 within the new framework does not account for sex or race and ethnicity, which may lead to misclassification of individuals without clinically meaningful excess adiposity. Future research should evaluate the incremental value of combining anthropometric measures and develop population‐specific thresholds to reduce misclassification of individuals without clinically meaningful obesity.

This study's primary strength is the use of nationally representative NHANES data to estimate trends in obesity prevalence under the Lancet framework. By integrating multiple anthropometric and functional measures, it provides the most comprehensive national estimates to date and highlights both the opportunities and challenges of implementing the Lancet Commission criteria in clinical practice and health policy.

Our study has some limitations. First, although the Excess Adiposity criteria distinguish preclinical from clinical obesity based on organ dysfunction or limitations in daily activities, retrospective NHANES data cannot fully establish whether these impairments are causally attributable to excess adiposity. Symptoms and functional limitations may arise from conditions not associated with obesity, which could lead to misclassification of clinical obesity. Future studies using longitudinal data will be essential to clarify the relationships between excess adiposity and specific organ dysfunctions, as recommended by the Lancet Commission.

Second, whole‐body DXA data were available only for adults aged ≤ 60 years and only during 2011–2016, and hip circumference data were collected beginning in 2017–2020. Because the Excess Adiposity criteria incorporate these measures, the changing availability of key data elements may have influenced temporal trends and contributed to the apparent acceleration in obesity prevalence in later cycles.

## 5 Conclusion

Obesity prevalence in the United States is substantially higher when the Excess Adiposity criteria is applied than when the BMI criteria alone are used, including nearly three out of five adults in 2021–2023. This new framework identifies many individuals with excess adiposity, organ dysfunction, and functional impairment and raises critical questions about how to target treatment and allocate resources as lifestyle interventions, pharmacotherapies, and metabolic surgeries become more widely available. Efforts to stratify risk and stage severity are needed to ensure that the expanded recognition of obesity translates into equitable, clinically meaningful, and sustainable prevention and treatment strategies.

## Author Contributions

J.H.H. had full access to all the data in the study and takes responsibility for the integrity of the data and the accuracy of the data analysis. Concept and design: all authors. Acquisition, analysis, or interpretation of data: all authors. Drafting of the manuscript: J.H.H. Critical review of the manuscript for important intellectual content: all authors. Statistical analysis: J.H.H. Administrative, technical, or material support: all authors. Supervision: N.L.

## Funding

Neda Laiteerapong was supported by funding from the National Institutes of Health (NIH) (P30 DK092949). Resources for this study were also provided in part by the University of Chicago Research Computing Center and the Center for Research Informatics. The funding organization had no role in the design and conduct of the study; collection, management, analysis, and interpretation of the data; preparation, review, or approval of the manuscript; and decision to submit the manuscript for publication.

## Conflicts of Interest

The authors declare no conflicts of interest.

## Supporting information


**Table S1:** Percentages of missing values from NHANES 1999–2023.
**Table S2:** Trends in population estimates and prevalence of US adults by Excess Adiposity criteria across BMI categories, NHANES 1999–2023.
**Table S3:** Sociodemographic, anthropometric, and clinical characteristics of adult NHANES participants by BMI and Excess Adiposity criteria for obesity, 1999–2023.

## Data Availability

All data utilized in the study are sourced from published literature and publicly accessible databases, all of which are cited in our manuscript.
