# Supplementary material for: Beyond BMI: The Impact of the New Lancet Commission Diagnostic Criteria on Prevalence of Obesity in the United States
Source: Obesity (Silver Spring). 2026 Feb 22;34(Suppl 1):29–42. doi: 10.1002/oby.70144 (PMC13250740; doi:10.1002/oby.70144)
Supplement: Supplementary file 1 — Table S1: Percentages of missing values from NHANES 1999–2023. Table S2: Trends in population estimates and prevalence of US adults by Excess Adiposity criteria across BMI categories, NHANES 1999–2023. Table S3: Sociodemographic, anthropometric, and clinical characteristics of adult NHANES participants by BMI and Excess Adiposity criteria for obesity, 1999–2023. [file OBY-34-29-s001.docx]

**Supplemental Online Content**

**Methods**

**Table S1. Percentages of Missing Values from NHANES 1999-2023**

**Table S2. Trends in Population Estimates and Prevalence of U.S. Adults by Excess Adiposity Criteria Across BMI Categories, NHANES 1999-2023**

**Table S3. Sociodemographic, Anthropometric, and Clinical Characteristics of Adult NHANES Participants by BMI and Excess Adiposity Criteria for Obesity, 1999-2023**

**References**

**Methods**

**1.1 Measure of organ dysfunction and daily activity limitations**

The new diagnostic framework defines two categories of obesity: preclinical and clinical obesity. Preclinical obesity was defined as a state of excess adiposity without current evidence of organ dysfunction or physical impairment but with an increased risk of developing obesity-related diseases. Clinical obesity was diagnosed based on presence of excess adiposity with (1) evidence of organ dysfunction due to obesity, or (2) substantial limitations in daily activities.

Measures of organ dysfunction and daily activity limitations were defined based on the Lancet Commission framework, which recognizes that obesity-related complications arise through multiple pathophysiological mechanisms, including excess adipose tissue mass, ectopic fat deposition, metabolic and inflammatory processes, and psychological factors.

Organ dysfunction was assessed across multiple systems, including respiratory, cardiovascular, metabolic, renal, urinary, hepatic, musculoskeletal, and reproductive systems. Respiratory dysfunction was defined as self- reported asthma or chronic obstructive pulmonary disease (COPD), and risk for obstructive sleep apnea was evaluated using the STOP-BANG questionnaire, with a score of ≥5 indicating elevated risk. Cardiovascular dysfunction encompassed self-reported diagnoses of angina, coronary heart disease (CHD), myocardial infarction (MI), congestive heart failure (CHF), or stroke, as well as symptoms such as exertional dyspnea or chest pain. Pulmonary hypertension (PAH) was identified using medication data Participants were classified as having PAH if they received a medication in the PAH drug class or if they were prescribed a PAH-specific agent, including bosentan or treprostinil. Metabolic dysfunction was defined as the presence of diabetes (HbA1c ≥6.5%, fasting glucose ≥125 mg/dL) or self-reported diabetes, or meeting established metabolic syndrome criteria, requiring at least three of the following: elevated triglycerides (≥150 mg/dL), low HDL (<50 mg/dL for females, <40 mg/dL for males), high blood pressure (≥130/85 mmHg), impaired glucose regulation (fasting glucose ≥100 mg/dL or HbA1c ≥5.7%), or increased WC (≥88 cm for females, ≥102 cm for males). Renal dysfunction was determined by the presence of microalbuminuria (albumin creatinine ratio [ACR] ≥30 mg/g) or chronic kidney disease (eGFR <60 mL/min/1.73m²). Urinary dysfunction was defined by self-reported urinary leakage. Metabolic dysfunction-associated steatotic liver disease (MASLD) required evidence of hepatic steatosis (CAP ≥263 dB/m), at least one metabolic risk factor, and exclusion of excess alcohol intake. Metabolic risk factors included elevated waist circumference, impaired glucose regulation (fasting glucose ≥100 mg/dL, HbA1c ≥5.7%, or diabetes), blood pressure ≥130/85 mm Hg or antihypertensive treatment, triglycerides ≥150 mg/dL or lipid-lowering treatment, or low HDL cholesterol (<50 mg/dL in females and <40 mg/dL in males). Fibrosis was defined using liver stiffness measurements with a cutoff of ≥8.6 kPa. Participants were classified as having MASLD with fibrosis if they met MASLD criteria and had liver stiffness values ≥8.6 kPa.^1-4^ Musculoskeletal dysfunction was assessed based on self-reported arthritis with arthritis-related limitations and/or a diagnosis of sarcopenia (only for NHANES 2011-2016), defined as low appendicular skeletal muscle mass (ASM) per height² according to the revised European Working Group on Sarcopenia in Older People (EWGSOP2) criteria (<7.26 kg/m² for males, <5.5 kg/m² for females). Reproductive dysfunction in females was identified based on self-reported infertility.

Limitation in daily activities was defined as having severe or some difficulty in performing any of the following activities: stooping, crouching, or kneeling; walking between rooms; standing up from an armless chair; getting in and out of bed; standing for long periods; or sitting for long periods.

**1.2 Handling Missing Data**

The missing data in this study was handled through a combination of recoding, and exclusion of cases with incomplete information to reduce non-response bias. Responses coded as “Refused” or “Don’t Know” were recoded as missing to ensure accurate analysis. Continuous variables such as blood pressure and cholesterol levels were aggregated using row means to minimize missingness while preserving available data. Missing variables were explicitly set to not available (n/a) before downstream analyses. The study did not perform multiple imputation but retained missing data in covariates to avoid introducing bias in prevalence estimates. Variables collected only in NHANES subsamples (e.g., fasting glucose, LDL-c, triglycerides) exhibited higher missingness due to survey design rather than participant nonresponse.

**Table S1. Percentages of Missing Values from NHANES 1999-2023 Analytic Samples**

|  | **1999-2004**  (N=12,752) | | **2005-2010**  (N=15,572) | | **2011-2016**  (N=15,708) | | **2017-2020**  (N=8,174) | | **2021-2023**  (N=5,847) | |
| --- | --- | --- | --- | --- | --- | --- | --- | --- | --- | --- |
|  | **N** | **%** | **N** | **%** | **N** | **%** | **N** | **%** | **N** | **%** |
| **Age** | 0 | 0 | 0 | 0 | 0 | 0 | 0 | 0 | 0 | 0 |
| **Female** | 0 | 0 | 0 | 0 | 0 | 0 | 0 | 0 | 0 | 0 |
| **Race** | 0 | 0 | 0 | 0 | 0 | 0 | 0 | 0 | 0 | 0 |
| **Education** | 24 | 0.2 | 23 | 0.1 | 11 | 0.1 | 11 | 0.1 | 3 | 0.1 |
| **Insurance status** | 161 | 1.3 | 13 | 0.1 | 21 | 0.1 | 18 | 0.2 | 9 | 0.2 |
| **Insurance types** | 2763 | 21.7 | 3799 | 24.4 | 3402 | 21.7 | 1368 | 16.7 | 610 | 10.4 |
| **Poverty ratio** | 1121 | 8.8 | 1270 | 8.2 | 1374 | 8.7 | 1130 | 13.8 | 761 | 13 |
| **Smoking status** | 15 | 0.1 | 9 | 0.1 | 18 | 0.1 | 4 | 0 | 6 | 0.1 |
| **Body mass index** | 0 | 0 | 0 | 0 | 0 | 0 | 0 | 0 | 0 | 0 |
| **Height** | 0 | 0 | 0 | 0 | 0 | 0 | 0 | 0 | 0 | 0 |
| **Hip circumference** | n/a | n/a | n/a | n/a | n/a | n/a | 317 | 3.9 | 270 | 4.6 |
| **Waist circumference** | 343 | 2.7 | 700 | 4.5 | 812 | 5.2 | 327 | 4 | 215 | 3.7 |
| **Weight** | 0 | 0 | 0 | 0 | 0 | 0 | 0 | 0 | 0 | 0 |
| **Total percent body fat** | n/a | n/a | n/a | n/a | 7269 | 46.3 | n/a | n/a | n/a | n/a |
| **Systolic blood pressure** | 583 | 4.6 | 691 | 4.4 | 530 | 3.4 | 776 | 9.5 | 158 | 2.7 |
| **Diastolic blood pressure** | 583 | 4.6 | 691 | 4.4 | 530 | 3.4 | 776 | 9.5 | 158 | 2.7 |
| **Fasting glucose**^b^ | 6788 | 53.2 | 8445 | 54.2 | 8495 | 54.1 | 4335 | 53 | 2735 | 46.8 |
| **Glycated hemoglobin (HbA1c)** | 550 | 4.3 | 834 | 5.4 | 698 | 4.4 | 407 | 5 | 268 | 4.6 |
| **Total cholesterol** | 735 | 5.8 | 924 | 5.9 | 888 | 5.7 | 557 | 6.8 | 521 | 8.9 |
| **Low-density lipoprotein** | 7335 | 57.5 | 8657 | 55.6 | 8836 | 56.3 | 4432 | 54.2 | n/a | n/a |
| **High-density lipoprotein** | 737 | 5.8 | 924 | 5.9 | 888 | 5.7 | 557 | 6.8 | 521 | 8.9 |
| **Triglycerides** | 6847 | 53.7 | 8495 | 54.6 | 8727 | 55.6 | 4400 | 53.8 | n/a | n/a |
| **Organ dysfunction**^a^ | | | | | | | | | | |
| **Diabetes** | 1 | 0 | 2 | 0 | 2 | 0 | 0 | 0 | 0 | 0 |
| **Hypertension** | 510 | 4 | 498 | 3.2 | 388 | 2.5 | 518 | 6.3 | 91 | 1.6 |
| **Dyslipidemia**^b^ | 6427 | 50.4 | 7565 | 48.6 | 5880 | 37.4 | 3024 | 37 | n/a | n/a |
| **Cardiovascular disease** | 7607 | 59.7 | 262 | 1.7 | 244 | 1.6 | 184 | 2.3 | 24 | 0.4 |
| **Coronary heart disease** | 79 | 0.6 | 69 | 0.4 | 57 | 0.4 | 25 | 0.3 | 25 | 0.4 |
| **Congestive heart failure** | 56 | 0.4 | 50 | 0.3 | 32 | 0.2 | 17 | 0.2 | 7 | 0.1 |
| **Myocardial infarction** | 25 | 0.2 | 32 | 0.2 | 15 | 0.1 | 12 | 0.1 | 9 | 0.2 |
| **Angina** | 8387 | 65.8 | 245 | 1.6 | 257 | 1.6 | 186 | 2.3 | n/a | n/a |
| **Stroke** | 14 | 0.1 | 30 | 0.2 | 13 | 0.1 | 13 | 0.2 | 11 | 0.2 |
| **Pulmonary hypertension**^c^ | 8386 | 65.8 | 0 | 0 | 0 | 0 | 0 | 0 | n/a | n/a |
| **Metabolic syndrome** | 0 | 0 | 0 | 0 | 0 | 0 | 0 | 0 | 0 | 0 |
| **MASLD with fibrosis** | n/a | n/a | n/a | n/a | n/a | n/a | 4903 | 60 | 3827 | 65.5 |
| **Chronic kidney disease** | 749 | 5.9 | 969 | 6.2 | 935 | 6 | 595 | 7.3 | n/a | n/a |
| **Asthma** | 11819 | 92.7 | 13547 | 87 | 13419 | 85.4 | 6913 | 84.6 | 4791 | 81.9 |
| **Sleep apnea** | n/a | n/a | 0 | 0 | 0 | 0 | 0 | 0 | n/a | n/a |
| **Depression** | 0 | 0 | 0 | 0 | 0 | 0 | 0 | 0 | 0 | 0 |
| **Osteoarthritis** | 10662 | 83.6 | 12820 | 82.3 | 12782 | 81.4 | 6297 | 77 | 4261 | 72.9 |
| **Urinary incontinence** | n/a | n/a | 1525 | 9.8 | 1551 | 9.9 | 502 | 6.1 | 793 | 13.6 |
| **Female infertility** | n/a | n/a | n/a | n/a | 4782 | 59.7 | 1669 | 40 | n/a | n/a |
| **Daily activity limitations** | 0 | 0 | 0 | 0 | 0 | 0 | n/a | n/a | n/a | n/a |

Abbreviations: n/a, not available; MASLD, metabolic dysfunction-associated steatotic liver disease.

^a^ Some composite health indicators (e.g., pulmonary hypertension, metabolic syndrome, sleep apnea, daily activity limitations) showed no missing values because they were derived from multiple component variables. Missing data in any component (e.g., blood pressure, waist circumference, triglycerides, glucose, or HbA1c) were handled through predefined rules, allowing calculation when partial information was available. Participants missing all relevant components were classified as missing.

^b^ Fasting glucose, LDL cholesterol, and triglyceride measurements were collected in subsamples of NHANES participants.

^c^ Medication use variables had higher missingness in earlier NHANES cycles due to lower response rates

**Table S2. Trends in Population Estimates and Prevalence of U.S. Adults by Excess Adiposity Criteria Across BMI Categories, NHANES 1999-2023**

**A. Obesity excluding body fat measures or WHR due to limited data availability**

|  | | | **Excess Adiposity criteria** | **1999-2004**  (N=12,752) | **2005-2010**  (N=15,572) | **2011-2016**  (N=15,708) | **2017-2020**  (N=8,174) | **2021-2023**  (N=5,847) | **P value** |
| --- | --- | --- | --- | --- | --- | --- | --- | --- | --- |
| **BMI Criteria, n in millions, (%)** | **Normal** | | **No obesity** | 58.1 (30.9) | 57.9 (28.2) | 53.1 (24.1) | 50.3 (21.7) | 53.3 (22.6) | **0.009** |
|  |  |  | **Obesity** | 4.8 (2.6) | 5.3 (2.6) | 7.1 (3.2) | 5.9 (2.5) | 6.2 (2.6) | 0.77 |
|  |  |  | **Preclinical** | 0.5 (0.3) | 0.8 (0.4) | 1.1 (0.5) | 0.8 (0.3) | 0.7 (0.3) | 0.52 |
|  |  |  | **Clinical** | 4.3 (2.3) | 4.5 (2.2) | 6.0 (2.7) | 5.1 (2.2) | 5.4 (2.3) | 0.59 |
|  | **Overweight** | | **No obesity** | 33.6 (17.9) | 34.8 (16.9) | 33.7 (15.3) | 33.7 (14.6) | 35.8 (15.2) | **0.04** |
|  |  |  | **Obesity** | 32.5 (17.3) | 35.1 (17.1) | 38.8 (17.6) | 38.0 (16.4) | 39.5 (16.7) | 0.29 |
|  |  |  | **Preclinical** | 4.0 (2.1) | 4.0 (2.0) | 5.4 (2.5) | 3.9 (1.7) | 5.0 (2.1) | 0.14 |
|  |  |  | **Clinical** | 28.5 (15.2) | 31.1 (15.1) | 33.4 (15.1) | 34.1 (14.7) | 34.5 (14.6) | **0.03** |
|  | **Obesity** | **All** | **No obesity** | 1.3 (0.7) | 2.7 (1.3) | 2.8 (1.3) | 2.4 (1.0) | 2.5 (1.0) | 0.17 |
|  |  |  | **Obesity** | 57.4 (30.6) | 69.5 (33.9) | 82.9 (37.6) | 98.3 (42.4) | 96.2 (40.8) | **0.007** |
|  |  |  | **Preclinical** | 5.3 (2.8) | 6.2 (3.0) | 8.0 (3.6) | 8.9 (3.9) | 8.6 (3.6) | 0.12 |
|  |  |  | **Clinical** | 52.2 (27.8) | 63.3 (30.8) | 74.9 (33.9) | 89.3 (38.6) | 87.6 (37.1) | **0.006** |
|  |  | **Class I** | **No obesity** | 0.7 (0.4) | 1.9 (0.9) | 2.3 (1.0) | 1.8 (0.8) | 2.0 (0.8) | 0.14 |
|  |  |  | **Obesity** | 34.3 (18.2) | 39.5 (19.2) | 47.3 (21.5) | 53.2 (23.0) | 50.4 (21.4) | 0.05 |
|  |  |  | **Preclinical** | 3.5 (1.9) | 4.3 (2.1) | 6.0 (2.7) | 6.5 (2.8) | 5.8 (2.5) | 0.26 |
|  |  |  | **Clinical** | 30.8 (16.4) | 35.2 (17.1) | 41.4 (18.8) | 46.8 (20.2) | 44.6 (18.9) | **0.02** |
|  |  | **Class II** | **No obesity** | 0.6 (0.3) | 0.9 (0.4) | 1.0 (0.4) | 1.1 (0.5) | 0.9 (0.4) | 0.22 |
|  |  |  | **Obesity** | 13.9 (7.4) | 17.7 (8.6) | 20.8 (9.4) | 26.2 (11.3) | 25.0 (10.6) | **0.009** |
|  |  |  | **Preclinical** | 1.2 (0.6) | 1.3 (0.6) | 1.5 (0.7) | 1.8 (0.8) | 1.7 (0.7) | **0.03** |
|  |  |  | **Clinical** | 12.7 (6.8) | 16.4 (8.0) | 19.3 (8.8) | 24.3 (10.5) | 23.3 (9.9) | **0.008** |
|  |  | **Class III** | **No obesity** | 0.0 (0.0) | 0.0 (0.0) | 0.0 (0.0) | 0.0 (0.0) | 0.0 (0.0) | 0.88 |
|  |  |  | **Obesity** | 9.3 (4.9) | 12.4 (6.0) | 16.4 (7.4) | 21.4 (9.2) | 22.9 (9.7) | **<0.001** |
|  |  |  | **Preclinical** | 0.5 (0.3) | 0.6 (0.3) | 0.9 (0.4) | 1.1 (0.5) | 1.4 (0.6) | 0.05 |
|  |  |  | **Clinical** | 8.7 (4.7) | 11.8 (5.7) | 15.5 (7.0) | 20.3 (8.8) | 21.5 (9.1) | **<0.001** |

**B. Obesity including body fat measures 2011-2016 and WHR 2017-2023**

|  | | | **Excess Adiposity criteria** | **1999-2004**  (N=12,752) | **2005-2010**  (N=15,572) | **2011-2016^a^**  (N=15,708) | **2017-2020^b^**  (N=8,174) | **2021-2023**  (N=5,847) | **P value** |
| --- | --- | --- | --- | --- | --- | --- | --- | --- | --- |
| **BMI Criteria, n in millions, (%)** | **Normal** | | **No obesity** | 58.1 (30.9) | 57.9 (28.2) | 43.7 (19.8) | 35.5 (15.3) | 36.9 (15.6) | **0.008** |
|  |  |  | **Obesity** | 4.8 (2.6) | 5.3 (2.6) | 16.5 (7.5) | 20.7 (8.9) | 22.6 (9.6) | 0.05 |
|  |  |  | **Preclinical** | 0.7 (0.4) | 0.8 (0.4) | 4.3 (2.0) | 2.9 (1.2) | 4.6 (1.9) | 0.18 |
|  |  |  | **Clinical** | 4.1 (2.2) | 4.5 (2.2) | 12.1 (5.5) | 17.8 (7.7) | 18.0 (7.6) | **0.04** |
|  | **Overweight** | | **No obesity** | 33.6 (17.9) | 34.8 (16.9) | 20.1 (9.1) | 12.2 (5.3) | 14.8 (6.3) | **0.02** |
|  |  |  | **Obesity** | 32.5 (17.3) | 35.1 (17.1) | 52.5 (23.8) | 59.5 (25.7) | 60.5 (25.6) | **0.03** |
|  |  |  | **Preclinical** | 5.3 (2.8) | 4.1 (2.0) | 9.9 (4.5) | 9.7 (4.2) | 10.8 (4.6) | 0.63 |
|  |  |  | **Clinical** | 27.2 (14.5) | 31.1 (15.1) | 42.6 (19.3) | 49.8 (21.5) | 49.7 (21.1) | **0.01** |
|  | **Obesity** | **All** | **No obesity** | 1.3 (0.7) | 2.7 (1.3) | 2.5 (1.1) | 2.4 (1.0) | 2.5 (1.0) | **0.15** |
|  |  |  | **Obesity** | 57.4 (30.6) | 69.5 (33.9) | 83.2 (37.7) | 98.3 (42.4) | 96.2 (40.8) | **0.008** |
|  |  |  | **Preclinical** | 7.0 (3.7) | 6.2 (3.0) | 8.2 (3.7) | 8.9 (3.9) | 8.6 (3.6) | 0.14 |
|  |  |  | **Clinical** | 50.5 (26.9) | 63.3 (30.8) | 75.1 (34.0) | 89.3 (38.6) | 87.6 (37.1) | **0.007** |
|  |  | **Class I** | **No obesity** | 0.7 (0.4) | 1.9 (0.9) | 1.9 (0.9) | 1.6 (0.7) | 1.9 (0.8) | 0.13 |
|  |  |  | **Obesity** | 34.3 (18.2) | 39.5 (19.2) | 47.7 (21.6) | 53.4 (23.0) | 50.4 (21.4) | 0.05 |
|  |  |  | **Preclinical** | 4.6 (2.5) | 4.3 (2.1) | 6.1 (2.8) | 6.5 (2.8) | 5.8 (2.5) | 0.29 |
|  |  |  | **Clinical** | 29.6 (15.8) | 35.2 (17.1) | 41.6 (18.8) | 46.9 (20.2) | 44.6 (18.9) | **0.02** |
|  |  | **Class II** | **No obesity** | 0.6 (0.3) | 0.9 (0.4) | 0.9 (0.4) | 1.1 (0.5) | 0.9 (0.4) | 0.26 |
|  |  |  | **Obesity** | 13.9 (7.4) | 17.7 (8.6) | 21.0 (9.5) | 26.2 (11.3) | 25.0 (10.6) | **0.008** |
|  |  |  | **Preclinical** | 1.5 (0.8) | 1.3 (0.6) | 1.5 (0.7) | 1.8 (0.8) | 1.7 (0.7) | **0.03** |
|  |  |  | **Clinical** | 12.4 (6.6) | 16.4 (8.0) | 19.4 (8.8) | 24.3 (10.5) | 23.3 (9.9) | **0.008** |
|  |  | **Class III** | **No obesity** | 0.0 (0.0) | 0.0 (0.0) | 0.0 (0.0) | 0.0 (0.0) | 0.1 (0.0) | 0.88 |
|  |  |  | **Obesity** | 9.3 (4.9) | 12.4 (6.0) | 16.4 (7.4) | 21.4 (9.2) | 22.9 (9.7) | **0.008** |
|  |  |  | **Preclinical** | 0.8 (0.4) | 0.6 (0.3) | 0.9 (0.4) | 1.1 (0.5) | 1.4 (0.6) | 0.05 |
|  |  |  | **Clinical** | 8.5 (4.5) | 11.8 (5.7) | 15.5 (7.0) | 20.3 (8.8) | 21.5 (9.1) | **0.007** |

Significant p-values are indicated in bold. P value for time trends.

^a^ Dual-energy x-ray absorptiometry (DEXA) total body fat measures were available for adults aged ≤60 years during 2011-2016.

^b^ Hip circumference measurements were available for the 2017-2023 NHANES cycle.

Abbreviations: WHR, waist to hip ratio

**Table S3. Sociodemographic, Anthropometric, and Clinical Characteristics of Adult NHANES Participants by BMI and Excess Adiposity Criteria for Obesity, 1999-2023**

**Obesity including body fat measures 2011-2016 and WHR 2017-2023**

|  | **1999-2004** | | **2005-2010** | | **2011-2016** | | **2017-2020** | | **2021-2023** | | **P value for BMI criteria** | **P value for Excess Adiposity criteria** |
| --- | --- | --- | --- | --- | --- | --- | --- | --- | --- | --- | --- | --- |
|  | **Obesity by BMI criteria** | **Obesity by Excess Adiposity criteria** | **Obesity by BMI criteria** | **Obesity by Excess Adiposity criteria** | **Obesity by BMI criteria** | **Obesity by Excess Adiposity criteria** | **Obesity by BMI criteria** | **Obesity by Excess Adiposity criteria** | **Obesity by BMI criteria** | **Obesity by Excess Adiposity criteria** |  |  |
| **Age, % (95% CIs)** | | | | | | | | | | | | |
| **Mean**  **(95% CIs)** | 47.3 (46.8-47.8) | 49.7 (49.3-50.2) | 48.0 (47.6-48.5) | 50.1 (49.8-50.5) | 48.7 (48.2-49.1) | 48.6 (48.2-49.0) | 48.8 (48.0-49.6) | 51.1 (50.5-51.7) | 49.8 (49.0-50.5) | 51.7 (51.1-52.3) | **0.003** | 0.43 |
| **20-44 yr** | 29 (27-30) | 40.7 (39.2-42.3) | 32.4 (31.1-33.7) | 43.8 (42.4-45.3) | 36.8 (35.3-38.2) | 65.3 (63.9-66.8) | 42.7 (40.2-45.2) | 66.4 (63.9-68.8) | 38.4 (35.8-41.0) | 64.5 (62.0-67.1) | **0.03** | 0.06 |
| **45-64 yr** | 35.8 (34.0-37.6) | 58.8 (56.9-60.7) | 39.4 (37.8-41.1) | 60.7 (59.0-62.4) | 42.1 (40.3-44.0) | 76.3 (74.7-78.0) | 45.4 (42.5-48.4) | 85.8 (83.7-88.0) | 48.2 (45.5-50.8) | 85.6 (83.8-87.4) | **0.01** | **0.01** |
| **≥65 yr** | 29.8 (27.9-31.7) | 62.7 (60.8-64.7) | 33.9 (32.1-35.7) | 65.0 (63.2-66.8) | 37.4 (35.2-39.6) | 67.7 (65.6-69.7) | 41.7 (38.6-44.8) | 88.9 (87.1-90.8) | 39.0 (36.6-41.5) | 87.8 (86.1-89.5) | **0.03** | **0.03** |
| **Sex , %(95% CIs)** | | | | | | | | | | | | |
| **Female** | 33.9 (32.5-35.3) | 59.3 (57.8-60.8) | 36.6 (35.2-37.9) | 61.5 (60.1-62.8) | 40.8 (39.4-42.3) | 75.7 (74.4-76.9) | 43.9 (41.7-46.1) | 77.0 (75.0-78.9) | 42.9 (40.8-45.0) | 76.3 (74.4-78.2) | **0.01** | 0.05 |
| **Male** | 28.6 (27.2-30.0) | 41.3 (39.8-42.8) | 33.8 (32.5-35.1) | 45.3 (43.9-46.7) | 36.7 (35.3-38.2) | 63.6 (62.2-65.1) | 43.0 (40.6-45.4) | 79.2 (77.1-81.2) | 40.7 (38.3-43.0) | 77.5 (75.4-79.5) | **0.01** | **0.01** |
| **Race/ethnicity, %(95% CIs)^a^** | | | | | | | | | | | | |
| Non-Hispanic White | 30.5 (29.2-31.7) | 51.2 (49.9-52.6) | 34.1 (32.9-35.3) | 54.4 (53.2-55.7) | 36.9 (35.5-38.3) | 69.3 (68.0-70.7) | 42.1 (39.7-44.5) | 78.7 (76.6-80.8) | 40.2 (38.3-42.2) | 77.4 (75.6-79.1) | **0.01** | **0.01** |
| Non-Hispanic Black | 41.4 (39.4-43.5) | 54.6 (52.5-56.7) | 47.0 (45.2-48.9) | 58.3 (56.5-60.2) | 48.5 (46.7-50.3) | 67.9 (66.2-69.5) | 50.8 (48.3-53.2) | 73.8 (71.5-76.0) | 52.1 (47.5-56.6) | 75.5 (71.4-79.6) | **0.02** | **0.004** |
| Hispanic | 31.3 (29.1-33.5) | 47.6 (45.2-50.0) | 36.7 (35.0-38.3) | 52.8 (51.0-54.6) | 44.3 (42.5-46.0) | 75.2 (73.7-76.8) | 45.7 (42.9-48.4) | 80.1 (77.8-82.4) | 44.3 (40.4-48.2) | 78.9 (75.4-82.4) | **0.03** | **0.04** |
| Non-Hispanic Asian | n/a | n/a | n/a | n/a | 24.3 (22.3-26.4) | 65.5 (63.3-67.7) | 33.0 (29.8-36.3) | 72.6 (69.5-75.8) | 28.6 (22.2-35.0) | 68.0 (61.3-74.7) | 0.42 | 0.47 |
| Other | 20.4 (16.3-24.6) | 37.1 (32.0-42.1) | 22.1 (18.6-25.6) | 35.7 (31.7-39.8) | 43.7 (37.9-49.6) | 68.3 (62.9-73.7) | 50.6 (43.4-57.9) | 79.6 (74.4-84.9) | 45.3 (39.0-51.5) | 77.2 (72.2-82.3) | **0.04** | **0.03** |
| **Education level,** **%(95% CIs)** | | | | | | | | | | | | |
| High school or less | 33.6 (32.2-35.0) | 53.8 (52.3-55.3) | 37.9 (36.5-39.2) | 56.7 (55.3-58.1) | 41.8 (40.2-43.3) | 72.6 (71.2-73.9) | 41.8 (40.2-43.3) | 72.6 (71.2-73.9) | 45.7 (43.0-48.4) | 80.4 (78.1-82.7) | **0.01** | **0.02** |
| Some college | 32.7 (30.8-34.6) | 51.9 (49.8-53.9) | 38.8 (37.0-40.5) | 56.5 (54.7-58.3) | 43.4 (41.5-45.2) | 72.9 (71.3-74.6) | 43.4 (41.5-45.2) | 72.9 (71.3-74.6) | 46.9 (44.1-49.8) | 79.1 (76.6-81.5) | **0.02** | **0.02** |
| College graduate | 25.1 (23.1-27.1) | 42.2 (39.9-44.5) | 26.7 (24.9-28.5) | 45.1 (43.1-47.2) | 30.7 (28.8-32.6) | 63.4 (61.4-65.3) | 30.7 (28.8-32.6) | 63.4 (61.4-65.3) | 33.3 (30.8-35.8) | 71.2 (68.7-73.6) | **0.009** | **0.02** |
| **Family poverty income (FPL) ratio, % (95% CIs)** | | | | | | | | | | | | |
| Less than 130% of FPL | 33.8 (31.7-35.8) | 53.0 (50.8-55.2) | 37.9 (36.2-39.6) | 55.7 (53.9-57.5) | 41.0 (39.3-42.7) | 70.2 (68.6-71.8) | 45.7 (42.6-48.8) | 78.9 (76.4-81.5) | 45.8 (42.0-49.5) | 76.5 (73.1-79.9) | **0.003** | **0.02** |
| 130%-349% of FPL | 33.1 (31.4-34.8) | 52.6 (50.8-54.5) | 37.1 (35.5-38.7) | 55.7 (54.1-57.4) | 41.7 (39.9-43.5) | 71.4 (69.8-73.0) | 48.0 (45.3-50.7) | 80.7 (78.6-82.8) | 44.4 (41.7-47.1) | 78.5 (76.1-80.9) | **0.02** | **0.02** |
| ≥350% of FPL | 28.4 (26.8-30.1) | 47.3 (45.5-49.1) | 32.9 (31.3-34.5) | 51.4 (49.7-53.1) | 35.4 (33.5-37.2) | 68.8 (67.0-70.6) | 40.7 (37.7-43.6) | 76.8 (74.2-79.4) | 38.0 (35.5-40.6) | 76.6 (74.3-78.8) | **0.02** | **0.01** |
| **Insurance status, % (95% CIs)** | | | | | | | | | | | | |
| No insurance | 28.9 (26.7-31.1) | 44.0 (41.6-46.5) | 31.9 (30.0-33.7) | 46.0 (44.0-48.0) | 38.9 (36.8-41.0) | 70.6 (68.7-72.6) | 39.2 (35.6-42.8) | 73.5 (70.0-76.9) | 43.1 (37.7-48.4) | 74.3 (69.4-79.1) | **0.01** | **0.05** |
| Any insurance | 31.8 (30.7-32.9) | 52.0 (50.8-53.2) | 36.0 (35.0-37.1) | 55.4 (54.3-56.5) | 38.8 (37.7-40.0) | 69.6 (68.5-70.7) | 44.1 (42.3-45.9) | 78.7 (77.2-80.3) | 41.6 (40.0-43.2) | 77.1 (75.7-78.6) | **0.02** | **0.02** |
| Medicaid | 40.7 (36.4-45.0) | 63.9 (59.8-68.1) | 47.1 (43.5-50.6) | 64.1 (60.7-67.5) | 48.0 (45.0-51.0) | 70.4 (67.7-73.1) | 48.6 (44.6-52.7) | 78.4 (75.2-81.7) | 48.3 (43.8-52.7) | 76.7 (72.6-80.7) | 0.13 | **0.03** |
| Medicare/Medi-Gap | 31.9 (29.8-34.0) | 63.5 (61.4-65.6) | 35.3 (33.4-37.2) | 66.0 (64.1-67.9) | 38.5 (36.1-40.9) | 70.0 (67.8-72.2) | 42.6 (39.4-45.8) | 89.2 (87.2-91.1) | 40.3 (37.7-42.8) | 87.7 (85.9-89.5) | **0.02** | **0.02** |
| Private | 31.0 (29.6-32.4) | 47.7 (46.3-49.2) | 34.9 (33.5-36.2) | 51.3 (49.9-52.8) | 37.5 (35.9-39.0) | 69.3 (67.8-70.8) | 43.4 (40.8-46.1) | 74.7 (72.3-77.0) | 40.1 (37.6-42.6) | 73.1 (70.8-75.3) | **0.02** | **0.03** |
| Other | 35.9 (29.0-42.8) | 60.0 (52.9-67.1) | 41.3 (37.1-45.6) | 60.0 (55.7-64.3) | 40.2 (36.3-44.1) | 70.3 (66.6-74.0) | 46.4 (40.4-52.3) | 77.6 (72.2-83.1) | 44.2 (38.4-50.0) | 73.5 (68.0-79.1) | 0.11 | 0.06 |
| **Smoking status, % (95% CIs)^b^** | | | | | | | | | | | | |
| Never smoker | 32.1 (30.7-33.5) | 50.6 (49.0-52.1) | 36.3 (35.0-37.6) | 53.4 (52.0-54.7) | 38.1 (36.8-39.4) | 69.6 (68.3-70.8) | 43.4 (41.3-45.6) | 75.9 (74.0-77.8) | 40.5 (38.5-42.5) | 73.9 (72.0-75.8) | **0.03** | **0.03** |
| Former smoker | 33.7 (31.7-35.7) | 56.3 (54.2-58.4) | 37.8 (35.9-39.7) | 59.7 (57.8-61.6) | 42.6 (40.4-44.8) | 73.0 (71.1-74.9) | 47.5 (44.0-50.9) | 84.3 (81.6-87.1) | 46.4 (43.3-49.5) | 83.5 (81.1-85.8) | **0.007** | **0.009** |
| Current smoker | 27.3 (25.4-29.3) | 44.3 (42.1-46.4) | 29.6 (27.8-31.5) | 47.1 (45.0-49.1) | 36.3 (34.0-38.5) | 66.5 (64.3-68.7) | 37.2 (33.5-40.9) | 75.7 (72.4-79.0) | 39.9 (35.8-43.9) | 78.4 (74.8-82.1) | **0.01** | **0.01** |
| **Anthropometric measures** | | | | | | | | | | | | |
| Mean weight, kg (95% CIs) | 100.9 (100.2-101.6) | 91.7 (91.1-92.3) | 101.8 (101.2-102.4) | 92.9 (92.4-93.5) | 101.4 (100.7-102.0) | 89.5 (88.9-90.0) | 102.6 (101.6-103.6) | 89.8 (88.9-90.6) | 102.5 (101.5-103.5) | 89.4 (88.6-90.2) | 0.09 | 0.16 |
| Mean BMI, kg/m2 (95% CIs) | 35.5 (35.3-35.7) | 32.3 (32.1-32.5) | 35.7 (35.5-35.9) | 32.7 (32.5-32.9) | 35.9 (35.7-36.1) | 31.6 (31.4-31.8) | 36.3 (36.0-36.6) | 31.8 (31.5-32.0) | 36.5 (36.2-36.8) | 31.7 (31.5-32.0) | **0.001** | 0.20 |
| Mean waist circumference, cm (95% CI) | 112.6 (112.2-113.1) | 107.1 (106.8-107.5) | 113.9 (113.5-114.3) | 108.2 (107.9-108.6) | 114.7 (114.3-115.2) | 105.8 (105.4-106.2) | 115.2 (114.5-115.8) | 106.1 (105.6-106.7) | 115.4 (114.7-116.0) | 106.0 (105.4-106.5) | **0.01** | 0.26 |
| **Cardiometabolic risk factors** | | | | | | | | | | | | |
| Mean systolic blood pressure, mmHg (95% CIs) | 127.1 (126.4-127.8) | 127.5 (126.9-128.1) | 124.7 (124.2-125.3) | 124.5 (124.0-124.9) | 125.6 (125.1-126.2) | 123.5 (123.1-123.9) | 123.2 (122.3-124.0) | 123.6 (123.0-124.3) | 122.6 (121.8-123.4) | 122.8 (122.2-123.4) | 0.06 | 0.14 |
| Mean diastolic blood pressure, mmHg (95% CIs) | 74.1 (73.6-74.6) | 73.2 (72.8-73.6) | 72.0 (71.6-72.4) | 71.2 (70.8-71.5) | 72.1 (71.7-72.5) | 71.3 (71.0-71.6) | 76.8 (76.3-77.4) | 75.3 (74.8-75.7) | 77.9 (77.4-78.5) | 76.1 (75.7-76.5) | 0.21 | 0.72 |
| Mean glucose, mg/dL (95% CIs) | 108.1 (106.2-110.1) | 106.9 (105.3-108.5) | 112.9 (111.2-114.6) | 110.4 (109.0-111.7) | 115.4 (113.5-117.4) | 110.3 (109.0-111.5) | 116.0 (113.5-118.6) | 112.8 (110.9-114.6) | 115.7 (113.0-118.5) | 112.4 (110.5-114.3) | 0.05 | 0.10 |
| Mean HbA1c, % (95% CIs) | 5.7 (5.7-5.8) | 5.7 (5.6-5.7) | 5.8 (5.8-5.8) | 5.7 (5.7-5.8) | 5.9 (5.9-5.9) | 5.7 (5.7-5.8) | 5.9 (5.8-5.9) | 5.8 (5.7-5.8) | 6.0 (5.9-6.0) | 5.8 (5.8-5.9) | **0.009** | 0.22 |
| Mean cholesterol, mg/dL (95% CIs) | 205.7 (204.0-207.3) | 208.1 (206.8-209.4) | 198.2 (196.8-199.5) | 200.8 (199.7-202.0) | 193.4 (192.0-194.9) | 195.0 (194.0-196.1) | 187.9 (185.9-190.0) | 190.1 (188.6-191.7) | 189.0 (186.9-191.1) | 191.0 (189.4-192.5) | **0.01** | **0.005** |
| Mean high density lipoprotein, mg/dL (95% CIs) | 46.4 (45.9-46.9) | 49.0 (48.6-49.5) | 47.0 (46.6-47.5) | 49.7 (49.3-50.1) | 48.0 (47.5-48.5) | 51.2 (50.8-51.6) | 48.4 (47.8-49.1) | 51.6 (51.0-52.2) | 49.3 (48.7-49.9) | 51.8 (51.2-52.3) | **0.001** | **0.03** |
| Mean low density lipoprotein, mg/dL (95% CIs) | 124.4 (122.4-126.5) | 124.9 (123.3-126.5) | 117.0 (115.3-118.7) | 118.8 (117.4-120.2) | 115.1 (113.4-116.9) | 116.1 (114.7-117.4) | 110.5 (107.9-113.0) | 112.5 (110.6-114.5) | n/a | n/a | **0.04** | **0.02** |
| **Chronic disease, % (95% CIs)** | | | | | | | | | | | | |
| Diabetes^c^ | 53.9 (50.9-56.9) | 75.9 (73.4-78.5) | 61.3 (58.9-63.6) | 78.7 (76.7-80.7) | 62.0 (59.6-64.4) | 84.4 (82.7-86.2) | 64.3 (60.8-67.7) | 93.6 (91.9-95.4) | 62.9 (59.6-66.2) | 91.9 (90.0-93.7) | 0.12 | **0.02** |
| Hypertension^d^ | 39.5 (38.0-40.9) | 62.1 (60.7-63.6) | 45.7 (44.3-47.1) | 66.9 (65.5-68.2) | 48.9 (47.4-50.4) | 76.7 (75.4-78.0) | 54.0 (51.7-56.2) | 89.0 (87.6-90.4) | 52.9 (50.9-55.0) | 87.7 (86.4-89.1) | **0.01** | **0.009** |
| Dyslipimidemia^e^ | 41.1 (39.6-42.7) | 63.5 (61.9-65.0) | 46.7 (45.3-48.1) | 68.0 (66.7-69.3) | 50.9 (49.3-52.4) | 80.8 (79.6-82.0) | 53.9 (51.6-56.3) | 90.1 (88.6-91.5) | n/a | n/a | **0.01** | **0.02** |
| Cardiovascular disease^f^ | 40.0 (36.8-43.1) | 65.6 (62.5-68.7) | 45.0 (42.2-47.8) | 70.8 (68.2-73.4) | 49.3 (46.1-52.5) | 70.4 (67.5-73.4) | 51.6 (46.9-56.3) | 90.6 (87.8-93.3) | 53.6 (49.2-58.1) | 89.5 (86.8-92.2) | **0.004** | **0.03** |
| Pulmonary hypertension^b^ | 33.0 (0.0-68.4) | 70.2 (35.7-100.0) | 24.4 (5.3-43.5) | 65.5 (44.4-86.6) | 32.7 (12.0-53.5) | 48.0 (25.8-70.1) | 49.7 (25.1-74.3) | 90.7 (76.5-100.0) | n/a | n/a | **0.22** | **0.34** |
| Metabolic syndrome^g^ | 58.2 (56.2–60.3) | 89.2 (87.9–90.4) | 64.0 (62.2–65.8) | 92.0 (91.0–93.0) | 67.4 (65.5–69.2) | 95.7 (94.9–96.5) | 70.9 (68.1–73.7) | 98.7 (98.1–99.4) | 67.6 (64.8–70.4) | 97.4 (96.5–98.3) | 0.06 | **0.04** |
| MASLD with fibrosis^h^ | n/a | n/a | n/a | n/a | n/a | n/a | 70.9 (68.1-73.7) | 98.7 (98.1-99.4) | 71.2 (65.8-76.6) | 93.0 (90.0-96.1) | n/a | n/a |
| Chronic kidney disease^i^ | 34.6 (30.8-38.5) | 65.5 (61.6-69.3) | 42.0 (38.7-45.3) | 69.6 (66.5-72.7) | 41.8 (38.0-45.5) | 68.6 (65.1-72.1) | 49.4 (43.9-55.0) | 88.9 (85.4-92.4) | n/a | n/a | 0.09 | 0.16 |
| Asthma^j^ | 42.1 (37.4-46.9) | 59.3 (54.5-64.0) | 48.9 (45.4-52.4) | 65.9 (62.6-69.3) | 48.6 (45.1-52.1) | 75.4 (72.4-78.4) | 54.0 (48.8-59.2) | 82.0 (78.0-86.0) | 50.4 (45.7-55.2) | 80.1 (76.3-83.9) | 0.14 | **0.02** |
| Sleep apnea^j^ | n/a | n/a | 73.5 (69.5-77.5) | 82.5 (79.0-85.9) | 73.4 (67.9-78.9) | 83.6 (79.0-88.2) | 72.9 (68.6-77.2) | 94.5 (92.3-96.7) | n/a | n/a | 0.20 | 0.19 |
| Depression^k^ | 34.2 (25.0-43.4) | 41.1 (31.5-50.6) | 44.2 (40.8-47.6) | 65.5 (62.2-68.7) | 50.5 (46.9-54.2) | 78.3 (75.3-81.3) | 51.6 (46.1-57.1) | 81.9 (77.7-86.1) | 47.8 (43.1-52.5) | 77.3 (73.4-81.2) | 0.25 | 0.17 |
| Osteoarthritis^b^ | 40.5 (36.9-44.1) | 67.8 (64.4-71.2) | 43.1 (39.8-46.3) | 69.7 (66.7-72.8) | 49.6 (46.5-52.7) | 76.9 (74.3-79.5) | 50.0 (45.4-54.5) | 88.7 (85.8-91.6) | 53.2 (49.6-56.9) | 89.7 (87.5-91.9) | **0.009** | **0.007** |
| Urinary incontinence^b^ | n/a | n/a | 43.6 (41.7-45.6) | 69.3 (67.5-71.1) | 47.4 (45.3-49.4) | 78.3 (76.6-80.0) | 52.6 (49.8-55.4) | 86.4 (84.5-88.3) | 47.2 (44.7-49.7) | 84.3 (82.5-86.1) | 0.36 | 0.10 |
| Female infertility^b^ | n/a | n/a | n/a | n/a | 53.7 (48.0-59.5) | 82.2 (77.8-86.6) | 49.7 (42.0-57.5) | 79.3 (73.0-85.6) | n/a | n/a | n/a | n/a |

Significant p-values are indicated in bold. P value for time trends.

^a^ Non-Hispanic Asian participants were not included in 1999-2004 because this group was not sampled prior to 2011-2012 cycle.

^b^ Self-reported

^c^ Diabetes is defined as a self-reported diagnosis, the use of diabetes medication, fasting plasma glucose (FPG) ≥125 mg/dL, or HbA1c ≥6.5%.

^d^ Hypertension is defined as a self-reported diagnosis, current treatment for hypertension, or an average systolic blood pressure ≥130 mmHg or diastolic blood pressure ≥80 mmHg.

^e^ Dyslipidemia is defined as a self-reported diagnosis, current treatment for hyperlipidemia, low-density lipoprotein cholesterol (LDL-C) ≥160 mg/dL, triglycerides ≥150 mg/dL, or high-density lipoprotein cholesterol (HDL-C) <40 mg/dL in men or <50 mg/dL in women.

^f^ Cardiovascular disease (CVD) history is defined as self-reported coronary heart disease, congestive heart failure, myocardial infarction, angina, or stroke, or prescription medication use for angina.

^g^ Metabolic syndrome is defined as meeting ≥3 of the following: abdominal obesity (waist circumference ≥88 cm for women or ≥102 cm for men), triglycerides ≥150 mg/dL, HDL-C <40 mg/dL in men or <50 mg/dL in women, blood pressure ≥130/85 mmHg or antihypertensive use, and fasting glucose ≥100 mg/dL or HbA1c ≥5.7%.

^h^ MASLD with fibrosis is defined as the presence of hepatic steatosis, indicated by a controlled attenuation parameter (CAP) ≥263 dB/m with at least one metabolic risk factor and liver stiffness ≥8.6 kPa, excluding participants with excess alcohol intake.^1-4^

^i^ Chronic kidney disease (CKD) is defined as estimated glomerular filtration rate (eGFR) <60 mL/min/1.73 m², calculated using the CKD-EPI 2009 equation.

^j^ Obstructive sleep apnea (OSA) is defined using the STOP-BANG score (snoring, tiredness, observed apneas, hypertension, BMI ≥35, age >50, and male sex), with a score ≥5 indicating OSA. Data are available for 2005-2006, 2007-2008, 2015-2016, and 2017-2020.

^k^ Depression is defined by meeting DSM-IV diagnostic criteria or a positive result on the PHQ-9 questionnaire.

Abbreviations: n/a, not available; WHR, waist to hip ratio; MASLD, metabolic dysfunction-associated steatotic liver disease.

**References**

1. Fujii H, Uchida-Kobayashi S, Kanamori A, et al. Defining optimal fatty liver index thresholds for MASLD and MetALD using controlled attenuation parameter as reference. *J Gastroenterol*. Oct 2025;60(10):1296-1309. doi:10.1007/s00535-025-02287-z

2. Kalligeros M, Vassilopoulos A, Vassilopoulos S, Victor DW, Mylonakis E, Noureddin M. Prevalence of Steatotic Liver Disease (MASLD, MetALD, and ALD) in the United States: NHANES 2017–2020. *Clinical Gastroenterology and Hepatology*. 2024/06/01/ 2024;22(6):1330-1332.e4. doi:<https://doi.org/10.1016/j.cgh.2023.11.003>

3. Sirli R, Sporea I. Controlled Attenuation Parameter for Quantification of Steatosis: Which Cut-Offs to Use? *Can J Gastroenterol Hepatol*. 2021;2021:6662760. doi:10.1155/2021/6662760

4. Lupșor-Platon M, Feier D, Stefănescu H, et al. Diagnostic accuracy of controlled attenuation parameter measured by transient elastography for the non-invasive assessment of liver steatosis: a prospective study. *J Gastrointestin Liver Dis*. Mar 2015;24(1):35-42. doi:10.15403/jgld.2014.1121.mlp
